# Meningioma: International Consortium on Meningiomas consensus review on scientific advances and treatment paradigms for clinicians, researchers, and patients

**DOI:** 10.1093/neuonc/noae082

**Published:** 2024-05-02

**Authors:** Justin Z Wang, Alexander P Landry, David R Raleigh, Felix Sahm, Kyle M Walsh, Roland Goldbrunner, Leeor S Yefet, Jörg C Tonn, Chloe Gui, Quinn T Ostrom, Jill Barnholtz-Sloan, Arie Perry, Yosef Ellenbogen, C Oliver Hanemann, Gerhard Jungwirth, Michael D Jenkinson, Ghazaleh Tabatabai, Tiit I Mathiesen, Michael W McDermott, Marcos Tatagiba, Christian la Fougère, Sybren L N Maas, Norbert Galldiks, Nathalie L Albert, Priscilla K Brastianos, Felix Ehret, Giuseppe Minniti, Katrin Lamszus, Franz L Ricklefs, Jens Schittenhelm, Katharine J Drummond, Ian F Dunn, Omar N Pathmanaban, Aaron A Cohen-Gadol, Erik P Sulman, Emeline Tabouret, Emelie Le Rhun, Christian Mawrin, Jennifer Moliterno, Michael Weller, Wenya (Linda) Bi, Andrew Gao, Stephen Yip, Maximilian Niyazi, The International Consortium on Meningiomas (ICOM), Kenneth Aldape, Patrick Y Wen, Susan Short, Matthias Preusser, Farshad Nassiri, Gelareh Zadeh

**Affiliations:** Princess Margaret Cancer Centre, University Health Network, Toronto, Ontario, Canada; Division of Neurosurgery, Department of Surgery, University of Toronto, Toronto, Ontario, Canada; MacFeeters Hamilton Neuro-Oncology Program, Princess Margaret Cancer Centre, University Health Network and University of Toronto, Toronto, Ontario, Canada; Princess Margaret Cancer Centre, University Health Network, Toronto, Ontario, Canada; Division of Neurosurgery, Department of Surgery, University of Toronto, Toronto, Ontario, Canada; MacFeeters Hamilton Neuro-Oncology Program, Princess Margaret Cancer Centre, University Health Network and University of Toronto, Toronto, Ontario, Canada; Department of Radiation Oncology, Neurological Surgery, and Pathology, University of California San Francisco, San Francisco, California, USA; Department of Neuropathology, University Hospital Heidelberg and German Consortium for Translational Cancer Research (DKTK), German Cancer Research Center (DKFZ), Heidelberg, Germany; Department of Neurosurgery, Duke University, Durham, North Carolina, USA; Center of Neurosurgery, Department of General Neurosurgery, University of Cologne, Cologne, Germany; Division of Neurosurgery, Department of Surgery, University of Toronto, Toronto, Ontario, Canada; Department of Neurosurgery, University Hospital Munich LMU, Munich, Germany; Princess Margaret Cancer Centre, University Health Network, Toronto, Ontario, Canada; Division of Neurosurgery, Department of Surgery, University of Toronto, Toronto, Ontario, Canada; MacFeeters Hamilton Neuro-Oncology Program, Princess Margaret Cancer Centre, University Health Network and University of Toronto, Toronto, Ontario, Canada; Duke Cancer Institute, Duke University School of Medicine, Durham, North Carolina, USA; Central Brain Tumor Registry of the United States, Hinsdale, Illinois, USA; Department of Neurosurgery, Duke University, Durham, North Carolina, USA; Center for Biomedical Informatics & Information Technology (CBIIT), National Cancer Institute, Bethesda, Maryland, USA; Trans Divisional Research Program (TDRP), Division of Cancer Epidemiology and Genetics (DCEG), National Cancer Institute, Bethesda, Maryland, USA; Central Brain Tumor Registry of the United States, Hinsdale, Illinois, USA; Department of Pathology, University of California San Francisco, San Francisco, California, USA; Princess Margaret Cancer Centre, University Health Network, Toronto, Ontario, Canada; Division of Neurosurgery, Department of Surgery, University of Toronto, Toronto, Ontario, Canada; MacFeeters Hamilton Neuro-Oncology Program, Princess Margaret Cancer Centre, University Health Network and University of Toronto, Toronto, Ontario, Canada; Peninsula Schools of Medicine, University of Plymouth University, Plymouth, UK; Division of Experimental Neurosurgery, Department of Neurosurgery, Heidelberg University, Heidelberg, Germany; Department of Neurosurgery, The Walton Centre NHS Foundation Trust, Liverpool, UK; Institute of Translational Medicine, University of Liverpool, UK; Department of Neurology and Interdisciplinary Neuro-Oncology, University Hospital Tübingen, Hertie Institute for Clinical Brain Research, Eberhard Karls University Tübingen, Tübingen, Germany; Cluster of Excellence (EXC 2180) “Image Guided and Functionally Instructed Tumor Therapies,” Eberhard Karls University Tübingen, Tübingen, Germany; Center for Neuro-Oncology, Comprehensive Cancer Center Tübingen-Stuttgart, University Hospital Tübingen, Tübingen, Germany; Department of Clinical Neuroscience, Karolinska Institute, Stockholm, Sweden; Department of Clinical Medicine, University of Copenhagen, Copenhagen, Denmark; Division of Neuroscience, Herbert Wertheim College of Medicine, Florida International University, Miami, Florida, USA; Miami Neuroscience Institute, Baptist Health of South Florida, Miami, Florida, USA; Department of Neurosurgery, University of Tübingen, Tübingen, Germany; Center for Neuro-Oncology, Comprehensive Cancer Center Tübingen-Stuttgart, University Hospital Tübingen, Tübingen, Germany; Nuclear Medicine and Clinical Molecular Imaging, University Hospital Tübingen, Germany; Cluster of Excellence (EXC 2180) “Image Guided and Functionally Instructed Tumor Therapies,” Eberhard Karls University Tübingen, Tübingen, Germany; Department of Pathology, Erasmus Medical Center, Rotterdam, The Netherlands; Department of Pathology, Leiden University Medical Center, Leiden, The Netherlands; Department of Neurology, Faculty of Medicine and University Hospital Cologne, University of Cologne, Cologne, Germany; Institute of Neuroscience and Medicine (IMN-3), Research Center Juelich, Juelich, Germany; Department of Nuclear Medicine, Ludwig Maximilians-University of Munich, Munich, Germany; Massachusetts General Hospital Cancer Center, Harvard Medical School, Boston, Massachusetts, USA; Department of Radiation Oncology, Charité - Universitätsmedizin Berlin, Corporate Member of Freie Universität Berlin and Humboldt-Universität zu Berlin, Berlin, Germany; Berlin Institute of Health, Charité - Universitätsmedizin Berlin, Berlin, Germany; Department of Radiological Sciences, Oncology and Anatomical Pathology, Sapienza University of Rome, Rome, Italy; Laboratory for Brain Tumor Biology, University Hospital Eppendorf, Hamburg, Germany; Department of Neurosurgery, University Medical Center Hamburg-Eppendorf, Hamburg, Germany; Department of Neuropathology, University Hospital Tübingen, Eberhard-Karls-University Tübingen, Tübingen, Germany; Center for Neuro-Oncology, Comprehensive Cancer Center Tübingen-Stuttgart, University Hospital Tübingen, Tübingen, Germany; Department of Neurosurgery, The Royal Melbourne Hospital, Melbourne, Victoria, Australia; Department of Neurosurgery, University of Oklahoma Health Sciences Center, Oklahoma City, Oklahoma, USA; Division of Neuroscience and Experimental Psychology, Manchester Centre for Clinical Neurosciences, Geoffrey Jefferson Brain Research Centre, University of Manchester, Manchester, UK; Department of Neurological Surgery, Indiana University, Indianapolis, Indiana, USA; Department of Radiation Oncology, NYU Grossman School of Medicine, New York, New York, USA; CNRS, INP, Inst Neurophysiopathol, Aix-Marseille University, Marseille, France; Department of Neurology & Brain Tumor Center, University Hospital and University of Zurich, Zurich, Switzerland; Department of Neuropathology, University Hospital Magdeburg, Magdeburg, Germany; Department of Neurosurgery, Yale School of Medicine, New Haven, Connecticut, USA; Department of Neurology and Brain Tumor Center, University Hospital and University of Zurich, Zurich, Switzerland; Department of Neurosurgery, Brigham and Women’s Hospital, Dana-Farber Cancer Institute, Harvard Medical School, Boston, Massachusetts, USA; Department of Laboratory Medicine and Pathobiology, University Health Network, Toronto, Ontario, Canada; Department of Pathology & Laboratory Medicine, University of British Columbia, Vancouver, British Columbia, Canada; Department of Radiation Oncology, University Hospital, Munich, Germany; German Cancer Consortium (DKTK), Munich, Germany; Bavarian Cancer Research Center (BZKF), Munich, Germany; Center for Neuro-Oncology, Comprehensive Cancer Center Tübingen-Stuttgart, University Hospital Tübingen, Tübingen, Germany; Center for Cancer Research, National Cancer Institute, Bethesda, Maryland, USA; Dana-Farber Cancer Institute, Brigham and Women’s Hospital and Harvard Medical School, Boston, Massachusetts, USA; Leeds Institute of Medical Research, St James’s University Hospital, Leeds, UK; Division of Oncology, Department of Medicine I, Medical University of Vienna, Vienna, Austria; Princess Margaret Cancer Centre, University Health Network, Toronto, Ontario, Canada; Division of Neurosurgery, Department of Surgery, University of Toronto, Toronto, Ontario, Canada; MacFeeters Hamilton Neuro-Oncology Program, Princess Margaret Cancer Centre, University Health Network and University of Toronto, Toronto, Ontario, Canada; Princess Margaret Cancer Centre, University Health Network, Toronto, Ontario, Canada; Division of Neurosurgery, Department of Surgery, University of Toronto, Toronto, Ontario, Canada; MacFeeters Hamilton Neuro-Oncology Program, Princess Margaret Cancer Centre, University Health Network and University of Toronto, Toronto, Ontario, Canada

**Keywords:** extra-axial, meningioma, methylation, molecular, neurofibromatosis 2, nonmalignant, radiotherapy

## Abstract

Meningiomas are the most common primary intracranial tumors in adults and are increasing in incidence due to the aging population and increased access to neuroimaging. While most exhibit nonmalignant behavior, a subset of meningiomas are biologically aggressive and are associated with treatment resistance, resulting in significant neurologic morbidity and even mortality. In recent years, meaningful advances in our understanding of the biology of these tumors have led to the incorporation of molecular biomarkers into their grading and prognostication. However, unlike other central nervous system (CNS) tumors, a unified molecular taxonomy for meningiomas has not yet been established and remains an overarching goal of the Consortium to Inform Molecular and Practical Approaches to CNS Tumor Taxonomy-Not Official World Health Organization (cIMPACT-NOW) working group. Additionally, clinical equipoise still remains on how specific meningioma cases and patient populations should be optimally managed. To address these existing gaps, members of the International Consortium on Meningiomas including field-leading experts, have prepared this comprehensive consensus narrative review directed toward clinicians, researchers, and patients. Included in this manuscript are detailed overviews of proposed molecular classifications, novel biomarkers, contemporary treatment strategies, trials on systemic therapies, health-related quality-of-life studies, and management strategies for unique meningioma patient populations. In each section, we discuss the current state of knowledge as well as ongoing clinical and research challenges to road map future directions for further investigation.

Meningioma is the most common primary intracranial tumor in adults. Historically, investigation into its molecular biology and pathogenesis has trailed other central nervous system (CNS) tumors. Since 2016, through the efforts of independent research groups and consortia including but not limited to the International Consortium on Meningiomas (ICOM) and the German Consortium on Aggressive Meningiomas (KAM), there has been a surge in molecular studies on meningiomas that have uncovered novel diagnostic and prognostic alterations. Despite these advances, meningioma treatments are still largely limited to surgery and radiotherapy (RT). Systemic medical therapies are reserved for otherwise treatment-refractory meningiomas in the context of clinical trials. There is a pressing need to translate findings from the current molecular era of meningioma research into meaningful improvements in decision-making and novel therapies. In this comprehensive consensus review, key advances in the understanding of meningioma biology will be discussed, with a focus on recent breakthroughs. Each section will also discuss ongoing controversies, critical knowledge gaps and areas of unmet need for clinicians, researchers, and patients that could be targeted for future research and investigation.

## Epidemiology and Risk Factors

**Figure 1.**
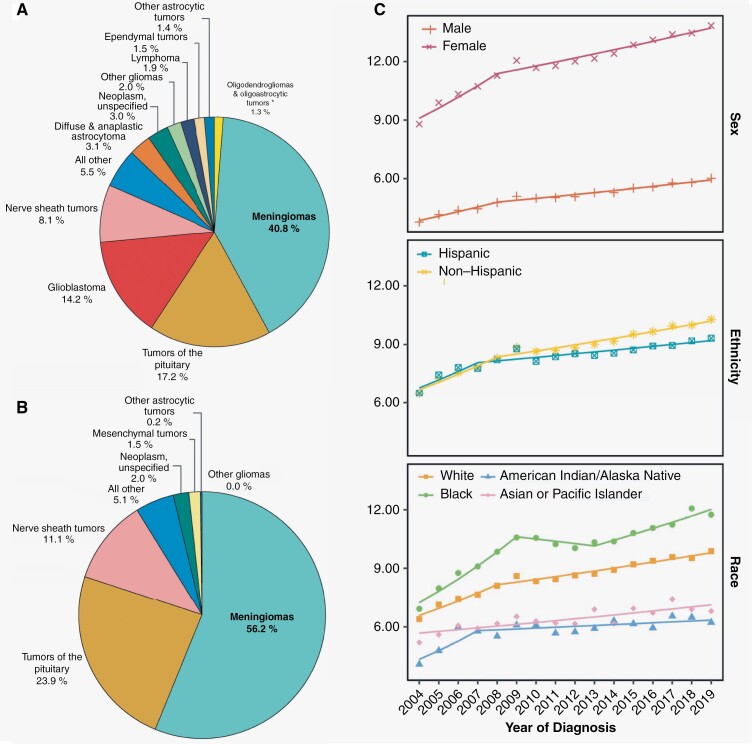

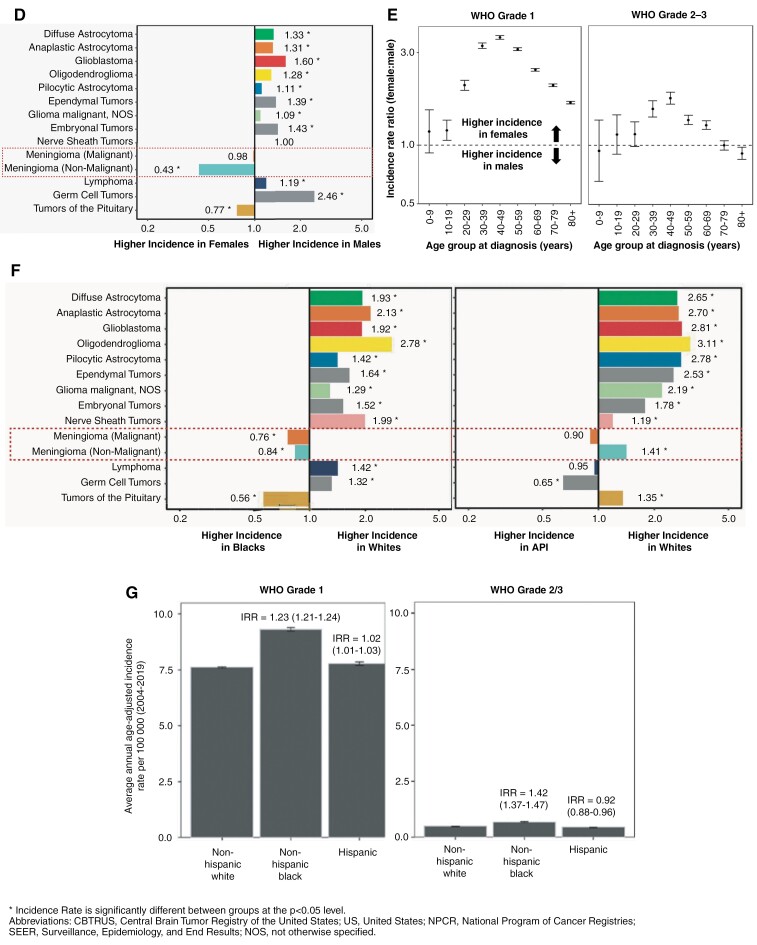
(A) Distribution of all primary brain tumors (malignant and nonmalignant combined; 5-year total = 453 623; annual average cases = 90 725) by histopathology. (B) Distribution of all nonmalignant primary brain tumors (5-year total = 326 894; average annual cases = 65 379) by histopathology. (C) Annual age-adjusted incidence rates of meningioma based on sex, ethnicity, and race. (D) Incidence rate ratios by sex (female-to-male) for selected primary brain and other central nervous system (CNS) tumor histopathologies with malignant and nonmalignant meningiomas highlighted. (E) Female-to-male incidence rate ratios and 95% confidence intervals (CI) for meningioma, by age group at diagnosis and stratified by WHO tumor grade. (F) Incidence rate ratios by race (white:black and white:Asian or Pacific Islander [API]) for selected primary brain and other CNS tumor histopathologies with malignant and nonmalignant meningiomas highlighted. (G) Average annual age-adjusted incidence rate and 95% confidence interval (CI) for meningioma by race/ethnicity and stratified by grade. Incidence rate ratios (IRR) and their 95% CI appear above bars and are calculated relative to non-Hispanic White individuals as the reference. Rates are age-adjusted to the 2000 US standard population. CBTRUS statistical report: US Cancer Statistics—National Program of Cancer Registries (NPCR) and the Surveillance, Epidemiology, and End Results (SEER), 2016–2020.^1,2^ Image panels A–D, F reused with permission from Ostrom et al. (2023).^1^ Image panels E, G reused with permission from Walsh et al. (2023).^2^ GBM, glioblastoma; CBTRUS, Central Brain Tumor Registry of the United States.

Meningiomas make up 40.8% of all primary brain tumors in the United States and 56.2% of “nonmalignant” primary brain tumors (Figure 1A and B).^1^ Incidence rates of nonmalignant meningioma are the highest amongst all CNS tumors at 9.73 per 100 000 population in the United States. These rates increase after the age of 65 years and again after the age of 85. Age-adjusted incidence rates of nonmalignant meningiomas continue to increase across different sexes, ethnicities, and races (Figure 1C). Meningiomas also account for the largest proportion of intradural spinal tumors in patients 20 years of age and older (39.9%), although spinal meningiomas represent only 4.2% of all diagnosed meningiomas.^1^

By World Health Organization (WHO) 2021 grading, 80.1% of reported meningiomas are CNS WHO grade 1, 18.3% are grade 2, and 1.5% are grade 3.^1,3^ “Nonmalignant” meningiomas (represented in the SEER database in the following proportions: 81.4% CNS WHO grade 1 and 18.4% CNS WHO grade 2) are 2.3 times more commonly diagnosed in females than in males, and this disparity is the largest between the ages of 35 and 44 (Figure 1D and E). Ten-year relative survival for nonmalignant meningioma is 83.4%. Although the SEER database quotes a 10-year survival rate of 60% for “malignant” meningiomas, this group is not exclusively comprised of CNS WHO grade 3 cases (63.6%) but also includes a sizeable proportion of CNS WHO grade 1 (20.4%) and grade 2 (15%) meningiomas. Notably, the designation of a “malignant” meningioma in this context is imprecisely defined and based on ICD coding instead of central neuropathological review. Non-registry data of exclusively CNS WHO grade 3 malignant or anaplastic meningiomas show far more dismal outcomes with a 5-year overall survival rate of 66% in one cohort and an estimated 10-year overall survival of only 14–24%.^1,2,4,5^

The incidence of intracranial meningiomas is higher in black patients compared to white patients and this disparity increases with higher tumor grade (Figure 1F and G).^2,6^ In turn, the incidence of “nonmalignant” meningiomas is higher in white patients compared to Asian-Pacific Islanders, although there may be a higher incidence of “malignant meningiomas” in the latter group (Figure 1F). The reasons behind these racial and ethnic differences remain unknown and the limitations of reporting based on population-based epidemiological data need to be considered, particularly for comparisons between different countries and continents.

Heritable genetic polymorphisms in *MLLT10* (MLLT10 histone lysine methyltransferase DOT1L cofactor) have also been robustly associated with increased meningioma risk.^7,8^ Distinct from germline variants that cause hereditary syndromes associated with meningiomas, *MLLT10* risk alleles are common at the population level and confer a comparatively modest increase in meningioma risk. These variants also increase the risk for ovarian cancer and estrogen receptor-positive breast cancer, and pan-cancer analyses implicate a potential estrogenic mechanism connecting *MLLT10* variation to the risk of diverse tumor types.^9^

**Table 1.** Recurrent Mutations Associated With Meningiomas

Gene	Location	Protein	Gene status	Frequency (%)	WHO grade	Modification	Associated meningioma phenotype	Effect of modification	Reference
***AKT1***	14q32.33	Protein kinase B alpha, beta, and gamma	Oncogene	10	1	Point mutation	Anterior/middle skull base location, NF2-wild-type meningiomas	Conformational change in protein, altering its localization from the cytoplasm to the plasma membrane, resulting in constitutive activation of the AKT1 kinase and activation of mTOR and ERK1/2 signaling pathways	^38,39,41,383–387^
***ARID1A***	1p36.11	AT-rich interaction domain 1A	Tumor suppressor	5	2, 3	Frameshift mutation	Recurrent and high-grade cases	Destabilizes SWI/SNF complex which normally modulates DNA accessibility for cellular processes involved in chromatin structure including transcription, DNA replication, and repair, resulting in global deregulation of gene transcription	^38,59–61,89^
***BAP1***	3p21.1	BRCA1-associated protein 1	Tumor suppressor	<1	2, 3	Splice site, nonsense, frameshift mutation	Rhabdoid and papillary histology	Impaired nuclear localization of a ubiquitin carboxy-terminal hydrolase that normally has tumor suppressor activity when bound to BRCA1 or BARD1	^38,45,53,62,70,71,388^
***BRAF* (V600E)**	7p34	B-Raf proto-oncogene	Oncogene	<1	3	Point mutation	Rhabdoid histology	Mimics phosphorylation of nearby serine and threonine residues resulting in BRAF activation and subsequent activation of the MAP kinase/ERK-signaling pathway	^43,93,389,390^
***CDH1***	16q22	E-cadherin	Tumor suppressor	30	1–3	Deletion, nonsense mutation	Unknown	Activation of wnt signaling via dysregulation of β-catenin and the APC protein, resulting in upregulation of cell cycling programs including c-myc and cyclin D1 pathways	^391–393^
***CDKN2A/B***	9p21.3	p16, p15^INK4b^	Tumor suppressor	1–5	3	Deletion	Anaplastic, biologically aggressive meningiomas	Loss of inhibition of CDK4 and CDK6 mediated phosphorylation of retinoblastoma family of proteins, leading to unchecked cell cycle progression from G1 to S-phase	^3,38,92,93,96,98,101,119,394–398^
***CDKN2C***	1p32.3	Cyclin-dependent kinase 4 inhibitor C (p18)	Tumor suppressor	1	2	Nonsense mutation	Atypical and anaplastic meningiomas	Activation of CDK4 and CDK6 resulting in loss of control of cell growth regulation and subsequent cell cycle G1 progression	^92,96^
***CHEK2***	22q12.1	Checkpoint kinase 2 (Chk2)	Tumor suppressor	50	1–3	Deletion, frameshift mutation	NF2-altered meningiomas	Defects in DNA homologous recombination or nonhomologous end-joining pathways following DNA damage including double-stranded DNA breaks resulting in increased chromosomal instability	^349,399^
***Dal-1***	18p11.3	Protein 4.1B	Tumor suppressor	50–80	1–3	Deletion, nonsense mutation	Multiple meningiomas, sporadic meningiomas	Dysregulation of cell-cell contact inhibition growth arrest normally mediated through actin cytoskeletal-associated proteins resulting in similar downstream effects as NF2 inactivation	^354,400–402^
***EZH2***	7q36.1	Enhancer of zeste homolog 2	Oncogene	1–5	2–3	Point mutation, amplification	Higher grade meningiomas	Dysregulation of catalytic subgroup of PRC2 complex, upregulation of proliferative genes in the cell cycle-retinoblastoma-E2F pathway	^403–405^
***FBXW7***	4q31.3	F-box and WD repeat domain containing 7	Tumor suppressor	1–5	1–3	Frameshift mutation	NF2-altered meningiomas	Deregulation of ubiquitin-mediated proteolysis of oncoproteins such as cyclin E, notch, c-Jun, c-Myc, mTOR resulting in increased tumorigenesis	^406,407^
***FGFR3***	4p16.3	Fibroblast growth factor receptor 3	Oncogene	15	1–2	Missense mutation	Skull base location, NF2-wild-type meningiomas	Increased mitogenic signaling from FGF receptors/kinases via activation of the PI3K-Akt-p70^S6K^ pathway and activation of STAT3	^408,409^
***KDM6A***	Xp11.3	Lysine demethylase 6A (UTX)	Tumor suppressor	5	2–3	Deletion	NF2-altered, higher-grade meningiomas	Dysregulation of polycomb repressive complex (PRC2) catalyzed histone methylation including of H3K27	^38,410^
***KLF4***	9p31	Krüppel-like factor 4	Tumor suppressor	10–15	1	Missense mutation	Skull base location, secretory meningiomas (when combined with *TRAF7* mutation)	Deactivation of CDKN1A (cyclin-dependent kinase inhibitor p21) resulting in cell proliferation, and loss of inhibition of CDK1 transcription	^10,39,45,132,411–415^
***NF2***	22q12.2	Merlin	Tumor suppressor	40–60	1–3	Deletion, nonsense mutation	Non-skull base location	Dysregulation of several essential cell proliferation and survival pathways including loss of cell-to-cell contact inhibition, activation of hippo pathway, mTOR/PI3K/AKT pathway, and receptor tyrosine kinases	^33–35,37,64,91,92,109,175,354,395,402,416–418^
***PIK3CA***	3q26.32	Phosphatidylinositol-4,5-bisphosphate 3-kinase, catalytic subunit alpha (p110α protein)	Oncogene	5	1	Point, missense mutation	Skull base location, benign WHO grade 1, progestin-related meningiomas	Activation of PI3 kinase and PI3K/AKT pathway resulting in downstream signaling cascades mediating cell survival. Apoptosis, transformation, metastasis, and cell migration	^42–44,419,420^
***POLR2A***	17p13.1	RNA polymerase II subunit A	Oncogene	5	1	Missense mutation	Skull base, tuberculum sellae location, benign WHO grade 1 meningiomas, meningothelial histology	Activation of catalytic subunit of RNA polymerase II, hijacking enzyme and driving cell proliferation and neoplastic progression	^40,45,415^
***PBRM1***	3p21.1	Protein polybromo-1	Tumor suppressor	1	3	Nonsense mutation, deletion	Papillary histology	Dysregulation of SWI/SNF chromatin remodeling complex, dysfunctional repair of DNA double-stranded breaks via ATM phosphorylation	^38,54^
***PTEN***	10q23.31	Phosphatase and tensin homolog	Tumor suppressor	2–6	2, 3	Frameshift mutation, deletion, rearrangement	Biologically aggressive, proliferative meningiomas	Dysregulation of AKT/PI3K pathway in the cell cytoplasm resulting from loss of feedback inhibition of AKT and subsequent uncontrolled cell cycle progression	^38,62–64^
***SMARCB1***	22q11.23	SWI/SNF-related matrix-associated actin-dependent regulator of chromatin subfamily B member 1	Tumor suppressor	<5	2, 3	Missense mutation	NF2-altered, atypical meningiomas	Inactivation of core subunit of SWI/SNF chromatin remodeling complex resulting in aberrant enhancer and promoter regulation and subsequent loss of transcriptional control	^61,421,422^
***SMARCE1***	17q21.2	SWI/SNF-related matrix-associated actin-dependent regulator of chromatin subfamily E member 1	Tumor suppressor	<1	1	Splice site, nonsense, frameshift mutation	Clear cell histology	Inactivation of subunit of SWI/SNF chromatin remodeling complex resulting in loss of apoptosis induction via tumor suppressor gene *CYLD* and other pathways	^47–51,69^
***SMO***	7p32.1	Smoothened	Oncogene	3–5	1	Point mutation	Anterior medial skull base location	Activation of the sonic hedgehog (SHH) signaling pathway resulting in cell proliferation, differentiation, and angiogenesis	^39,41,42,387,423,424^
***SUFU***	10q24.32	Suppressor of fused homolog	Tumor suppressor	1–2	2–3	Frameshift mutation	Familial multiple meningiomas	Abnormal constitutive upregulation of downstream Gli-mediated transcription factors in SHH pathway	^74,75,425,426^
***TERTp***	5p15.33	Telomerase reverse transcriptase promoter	Oncogene	5.5	3	Point mutation	Biologically aggressive, high-grade meningiomas	Activation of telomerase-mediated telomere stabilization resulting in delayed replicative senescence and increased telomere-driven genomic instability	^56–59,427–429^
***TRAF7***	16p13.3	TNF receptor-associated factors 7	Tumor suppressor	20–25	1	Missense mutation	Skull base location, brain invasion	Disruption of catalytic activity of E3 ubiquitin ligase interaction with MAPK pathway and RAS GTPases, altering actin dynamics and promoting anchorage-independent growth	^39,132,430^

While some genes and their alterations have been well investigated by multiple independent groups, others may have been identified in only single or small cohorts of meningiomas. Consequently, the overall frequencies of less studied mutations may be confounded by smaller cohorts biased to include either more benign or clinically aggressive cases from which the data is obtained.

Despite progress in identifying exogeneous and endogenous factors associated with risk of meningioma development, relatively few modifiable risk factors have been definitively identified. These few include ionizing radiation, elevated body mass index, methotrexate treatment, and cigarette smoking (where the increased risk is restricted to male sex only).^10–21^ The most well-validated of these risk factors is cranial irradiation, with a linear dose–response association between the radiation dose received and the risk of subsequent meningioma development, particularly in patients who were treated under the age of 10 (to be further discussed in the *Radiation-Induced Meningiomas [RIMs]* section later in the article).^19,20^ Despite the fact that meningiomas are known to commonly express progesterone receptors, estrogen receptor expression is rare and there are conflicting results on the risk of meningioma growth or development in response to endogenous and exogenous sex hormones.^22–28^ Several large retrospective studies have demonstrated a positive association between current or past use of hormone replacement therapy and the diagnosis of a meningioma.^21,29^ On a population-level, pregnancy does not appear to be a risk factor for meningioma development, although accelerated growth of an existing meningioma during pregnancy has long been described.^30–32^There is currently insufficient evidence to support a standardized screening approach such as germline genetic testing or routine neuroimaging, even in higher risk cohorts such as female relatives of meningioma patients with the *MLLT10* risk allele, or women on hormone replacement therapy. This remains an area of active investigation and guidelines may evolve with emerging evidence.

## Genomics and Biology

The neurofibromatosis-2 (*NF2*) gene was the first gene to be implicated in meningioma development (Table 1). It remains the most common genetic abnormality in sporadic meningiomas, inclusive of short structural or copy number variants, and is found in up to 60% of all meningioma cases. As a tumor suppressor gene on chromosome 22q12.2, *NF2* encodes the protein Merlin which has been implicated in the inhibition of signals from the PI3K/Akt, Raf/MEK/ERK, and mTOR signaling pathways in non-meningioma cells.^33–35^ In meningioma cells, loss of Merlin may also be associated with overexpression of yes-associated protein 1 (*YAP1*) and deregulation of the Hippo signaling pathway, leading to increased cell proliferation and anchorage-independent growth.^36^ NF2/Merlin loss may also increase the apoptotic threshold of meningioma cells and decrease susceptibility to cytotoxic therapies through interferon regulatory factor-mediated gene expression pathways.^37^ Consequently, meningiomas with *NF2* alterations have an increased risk of being higher grade and more biologically aggressive, although benign *NF2*-mutant cases are still observed. The rate of *NF2* mutations in meningiomas in one large study were found to be 37% in CNS WHO grade 1 cases (81/220), 60% of grade 2 cases (265/441), and 69% of grade 3 tumors (122/176).^38^

More recently, recurrent mutations in *TRAF7* (tumor necrosis factor receptor-associated factor 7), *KLF4* (Kruppel-like factor 4), *AKT1* (AKT serine/threonine kinase 1), *SMO* (Smoothened), *SUFU* (Suppressor of fused homolog), *PRKAR1A* (protein kinase cAMP-dependent type I regulatory subunit alpha)*, PIK3CA* (phosphatidylinositol-4,5-bisphosphate 3-kinase catalytic subunit alpha), and *POLR2A* (RNA Polymerase II Subunit A) have been discovered in meningiomas without any *NF2* alterations (Table 1).^39–44^ Compared to *NF2*-altered meningiomas, meningiomas with these “non-*NF2*” mutations tend to be lower WHO grade, have fewer chromosomal abnormalities, and generally have better clinical outcomes with standard therapies.

The anatomic location of meningiomas also appear to have genomic underpinnings. Meningiomas with *NF2* loss tend to be located along the cerebral convexities or in the posterior/lateral skull base. Those with non-*NF2* mutations (eg, *TRAF7*, *SMO, SUFU, and PRKAR1A*) are more common around the anterior skull base. Meningiomas with combined *NF2/SMARCB1* mutations (2 genes in close physical proximity to one another on chromosome 22q) may be more commonly found along the anterior falx.^39,45^ Alterations in several meningioma driver genes (including *NF2* and *TRAF7*) have also been found in normal leptomeninges with similar anatomic predilection.^46^

Mutations in *SMARCE1* (SWI/SNF-related matrix-associated actin-dependent regulator of chromatin subfamily B member 1)*, BAP1* (BRCA1 associated protein-1), and *PBRM1* are associated with different meningioma histologic subtypes. *SMARCE1* loss is found in almost all clear cell meningioma, which are currently CNS WHO grade 2 by the 2021 classification.^47–51^*SMARCE1* encodes for a protein involved in the SWI/SNF chromatin remodeling complex and consequently *SMARCE1*-deficient cells may be susceptible to SWI/SNF inhibition.^50^ Inactivation of *BAP1* has been associated with rhabdoid and papillary histology and is almost universally associated with poor prognosis, although fewer than 30 of these cases have been reported in the literature.^52,53^*PBRM1* mutations often co-occur with *BAP1* mutations and are associated with papillary or sometimes rhabdoid histology.^54^ Notably, alterations in *SMARCE1* and *BAP1* appear to be independent of *NF2* mutation or loss, identifying a small, rare group of *NF2-*wild-type meningiomas that are unusually aggressive.

Finally, mutations in the *TERT* (telomerase reverse transcriptase) promoter (*TERT*p) have been added to the most recent iteration of the WHO classification as an independent marker of grade 3 meningiomas.^3^ While rare in meningiomas, this alteration is associated with significantly worse progression-free survival (PFS) and overall survival when present.^55,56^*TERT* functions to maintain DNA telomere ends, resulting in immortalization of cancer cells. Successful downstream blockade of *TERT*p activity via E26 transformation-specific (ETS) transcription factor inhibition is a potential therapeutic strategy for these tumors.^57,58^ Other rare mutations associated with higher-grade meningiomas include *ARID1A*, *PTEN*, and *PBRM1*.^38,54,59–64^

Some of these recurrent mutations identified in meningiomas may occur in the germline and correspond with hereditary meningioma syndromes. The most common of these is germline mutation of *NF2* resulting in what was historically referred to as syndromic Neurofibromatosis type 2, an autosomal dominant condition characterized by the growth of multiple schwannomas and meningiomas.^65^ Due to the overlapping phenotypes of Neurofibromatosis type 2 and schwannomatosis, the latter being a tumor predisposition syndrome also characterized by the development of multiple schwannomas, the diagnostic criteria and disease nomenclature for NF2 and schwannomatosis was updated in 2022. “Schwannomatosis” is now an umbrella term referring to the phenotype of multiple schwannomas, and the individual syndromes are named by their underlying genetic mutation. The previously defined "NF2 syndrome" has now been renamed "*NF2*-related schwannomatosis" (NF2-SWN) and this is the term which will be used in the rest of this article. Schwannomatosis is designated as *SMARCB1*-related, *LZTR1*-related, or 22q-related.^66^ Meningiomas are uncommon in non-*NF2*-related schwannomatosis and not part of the diagnostic criteria of *LZTR1*-related schwannomatosis and *SMARCB1-*related schwannomatosis despite the presence of *SMARCB1* mutations in sporadic clear cell meningiomas.^67^ Meningiomas in NF2-SWN patients will be discussed in more detail in a later section.

Other hereditary syndromes associated with meningiomas are less common and there is an overall lack of data to support these germline mutations driving meningioma tumorigenesis. A rare autosomal dominant inheritance pattern of *SMARCE1* mutations predisposing to intracranial and spinal meningiomas with clear cell histology has been reported.^68,69^ Germline *BAP1* loss causes a hereditary cancer predisposition syndrome phenotypically associated with mesothelioma and uveal melanoma. As mentioned above, sporadic and hereditary germline *BAP1* mutations have been linked to the development of rhabdoid and papillary meningiomas in small case series, which may also have an increased risk of extracranial metastasis.^53,70–73^ Other meningioma-associated tumor predisposition syndromes include: Werner syndrome, an autosomal recessive condition caused by biallelic loss of *WRN*, characterized by premature aging; Gorlin syndrome (or familial multiple meningiomas), an autosomal dominant condition resulting from germline mutations in Sonic Hedgehog (Hh) pathway genes including *PTCH1* or *SUFU,* characterized by multiple basal cell carcinomas and biologically aggressive meningiomas^74–76^; and Cowden syndrome, another autosomal dominant condition resulting from germline *PTEN* mutation, characterized by multiple cancers including breast and thyroid.^62^ Notably, these are all rare entities and only a subset of patients with each syndrome will develop meningiomas. The overall prevalence of these syndromes is estimated to be between 1 in 20 000 (in some Japanese populations) to as few as 1 in 1 000 000 for Werner syndrome, between 1 in 30 000 to 1 in 250,000 for Gorlin syndrome, and between 1 in 200 000 to 1 in 250 000 for Cowden syndrome.^77–79^ The specific prevalence of germline *SMARCE1* and *BAP1* mutations is less clear given their rarity.

In addition to single-gene alterations, somatic copy number alterations (other than loss of 22q) have also been implicated in meningioma development (Table 2). Deletions of chromosome arm 1p were identified early in meningiomas, where it was associated with significantly shorter progression free survival (PFS).^80–86^ Multiple genomic targets of 1p loss have been proposed including *CDKN2C*, *RAD54*, *EPB41*, *GADD45A*, *ALPL*, *MUTYH*, *PRDX1*, *FOXD2*, *FOXE3*, and *PTCH2*, but their independent prognostic contributions to a more aggressive meningioma phenotype remain relatively unknown and remains an area of study study.^87,88^ Losses of chromosomal arms 6p, 10q, 14q, 18q, and gains of 17q and 20q were found to be recurrent across high-grade meningiomas and additional studies have also linked losses of 4q, 6, and 19p with poorer PFS (Table 2).^89–94^ In cases without chromosome 22q loss, several unique somatic copy number alterations including those affecting chromosomes 2q and 7q were found to be associated with dysregulated Hh signaling activation in otherwise mutation-negative meningiomas.^95^

**Table 2.** Recurrent Copy Number Alterations Observed in Meningiomas and Their Association With Clinical Prognosis (When Known)

Chromosome arm/gene	Loss/gain	Approximate frequency in all meningiomas	Associated clinical prognosis
1p	Loss	30%–50%	Intermediate to poor
1q	Gain	5%	Poor
3p	Loss	10%–15%	Intermediate
4p/q	Loss	5%–10%	Intermediate to poor
5p/q	Gain	2%–3%	Good
6q	Loss	15%–20%	Poor
7p	Loss	<5%	Intermediate-poor
8p	Gain	<5%	Unknown
10q	Loss	10%	Poor
11q	Loss	5%	Intermediate
12p/q	Gain	2%–3%	Good
14q	Loss	20%	Poor
15q	Gain	<5%	Unknown
16q	Gain	5%	Unknown
17q	Gain	5%–10%	Unknown
18q	Loss	15%–20%	Poor
20q	Gain	10%	Unknown
22q	Loss	50%–60%	Intermediate to poor

Importantly, homozygous loss of the *CDKN2A/B* (cyclin-dependent kinase inhibitor 2A/B) locus on chromosome 9p21 was incorporated into the 2021 WHO classification as a defining feature of CNS WHO grade 3 meningioma.^3^*CDKN2A/B* encodes for multiple tumor suppressor proteins including p16, which inhibits the G1-to-S transition in the cycle cell through the inactivation of CDK4 and CDK6. Its loss has been implicated in dysregulated cell cycle progression in multiple cancers.^96,97^ In meningioma, homozygous deletion of *CDKN2A/B* is associated with significantly shorter PFS, and even heterozygous deletions have been found to be associated with similarly poor outcomes in some studies.^98–101^ In meningiomas with an intact *CDKN2A/B* locus, higher mRNA expression of *CDKN2A* was also associated with significantly shorter PFS and increased rates of resistance to CDK inhibitors.^98^

The integration of prognostic copy number alterations with contemporary histological grading has resulted in the development of “integrated” or “molecular-morphologic” grading schemes. For example, a nomogram was developed whereby one point is assigned to each of the following copy number alterations if present: 1p-, 3p-, 4p/q-, 6p/q-, 10p/q-, 14q-, 18p/q-, 19p/q-, *CDKN2A/B*- in addition to one point for 4–19 mitoses per 10 high-powered fields or 2 points for more than 20 mitoses. A total of 0–1 points in this proposed grading paradigm would constitute an "Integrated grade 1" meningioma, 2–3 points for an "Integrated grade 2" case, and 4 or more points for an "Integrated grade 3" case. This integrated grading system was able to predict tumor recurrence/progression more accurately than standard WHO grading alone.^92^ Similar models have been developed by assigning scores based on combining WHO grade (histologic grade), DKFZ methylation-class family (benign, intermediate, or malignant; to be described further below), and the presence of 3 prognostic CNVs: 1p-, 6q-, and/or 14q-. This “integrated molecular morphologic risk” also had significantly better accuracy for outcome prediction compared to WHO grade or any of these molecular criteria alone, particularly for meningiomas bordering the threshold between CNS WHO grades 1 and 2.^93^

## Histopathologic Classification

**Figure 2.**
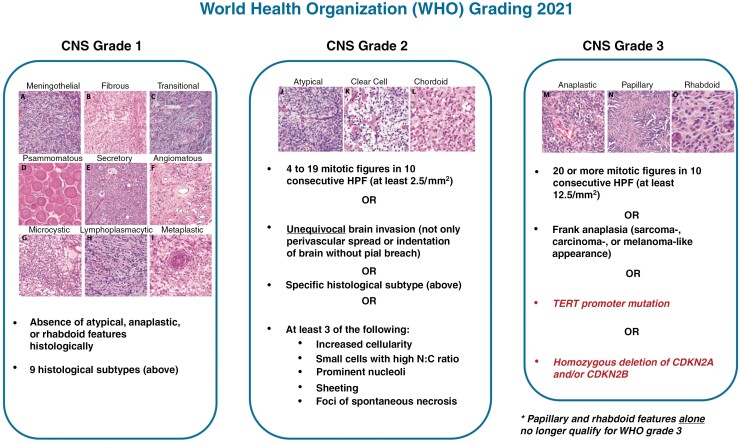
Updated 2021 World Health Organization (WHO) Grading criteria for meningiomas including histological subtypes for CNS WHO grade 1 cases: (A) meningothelial, (B) fibrous, (C) transitional, (D) psammomatous, (E) secretory, (F) angiomatous, (G) microcystic, (H) lymphoplasmacyte-rich, (I) metaplastic; CNS WHO grade 2 cases: (J) atypical, (K) clear cell, (L) chordoid; and CNS WHO grade 3 cases: (M) anaplastic, (N) papillary, (O) rhabdoid. HPF- high-powered fields; N:C, nuclear to cytoplasm. Histological image panels (A–O) used with permission from Bi et al. (2016).^91^ CNS, central nervous system; HPF, high-powered fields; N:C, nuclear to cytoplasm; TERT, telomerase reverse transcriptase.

The histopathologic characteristics of meningioma have been the main correlate to outcome for decades and still form the basis of contemporary WHO grading. Released in 2021, the 5th edition of the WHO CNS classification is the first to include molecular criteria for the definition of a CNS WHO grade 3 meningioma: presence of a *TERT*p hotspot mutation or homozygous loss of *CDKN2A/B.* These molecular alterations are rare in meningiomas, particularly in cases that do not have other worrisome histologic findings. In the absence of these alterations, which automatically impart a CNS WHO grade 3 designation, grading is assigned based on histopathologic features such as the number of mitotic figures or identification of at least 3 out of 5 “soft” criteria for atypia (sheeting architecture, hypercellularity, small cell formation, macronucleoli, spontaneous necrosis; Figure 2).^3,102^

While the presence of brain invasion alone is now sufficient for a designation of CNS WHO grade 2 meningioma, its association with outcome in the absence of any other higher grade histopathological features (eg, brain invasion without elevated mitotic index, hypercellularity, loss of architecture, small cell change, spontaneous necrosis, or prominent nucleoli) remains unclear.^103–105^ Given that cases of brain invasion alone as a solitary atypical finding is rare, only a minority of meningioma cases will likely require retrospective re-grading based solely on this feature.^104^ More work is needed to understand the biological significance and mechanism of brain invasion in meningioma.^106^ Additionally, current intraoperative sampling methods to identify brain invasion vary significantly between neurosurgical departments worldwide and this too requires standardization given that many pathology samples for this extra-axial tumor may lack brain tissue altogether.^107,108^ A systematic, structured method of safely sampling areas suspicious for brain invasion during surgery may be needed to optimize the diagnostic yield for detecting CNS invasion.

While chordoid or clear cell histology still mandates a CNS WHO grade 2 classification by the 2021 criteria, rhabdoid or papillary histology alone without other features of anaplasia or malignancy are now insufficient to render a CNS WHO grade 3 designation (Figure 2).^3,102^

## Biomarkers and Molecular Classification

Given the prognostic alterations uncovered in meningiomas, significant efforts have been made to develop a unified molecular classification system similar to those that exist for glioma and medulloblastoma.^3^ In 2017, the first landmark studies on DNA methylation-based classification systems for meningioma were published. These models were capable of stratifying meningiomas into groups at high- or low-risk of recurrence/progression and further identified 6 unique methylation-defined subgroups of meningioma (benign-1, benign-2, benign-3, intermediate-A, intermediate-B, and malignant) that appeared to reflect tumor biology more accurately than WHO grade alone.^109,110^ The DNA methylation profiles of meningiomas could be further combined with prognostic clinical variables including histologic grade and extent of resection to robustly predict clinical outcome and help guide decisions on adjuvant treatment after surgery.^111^

**Figure 3.**
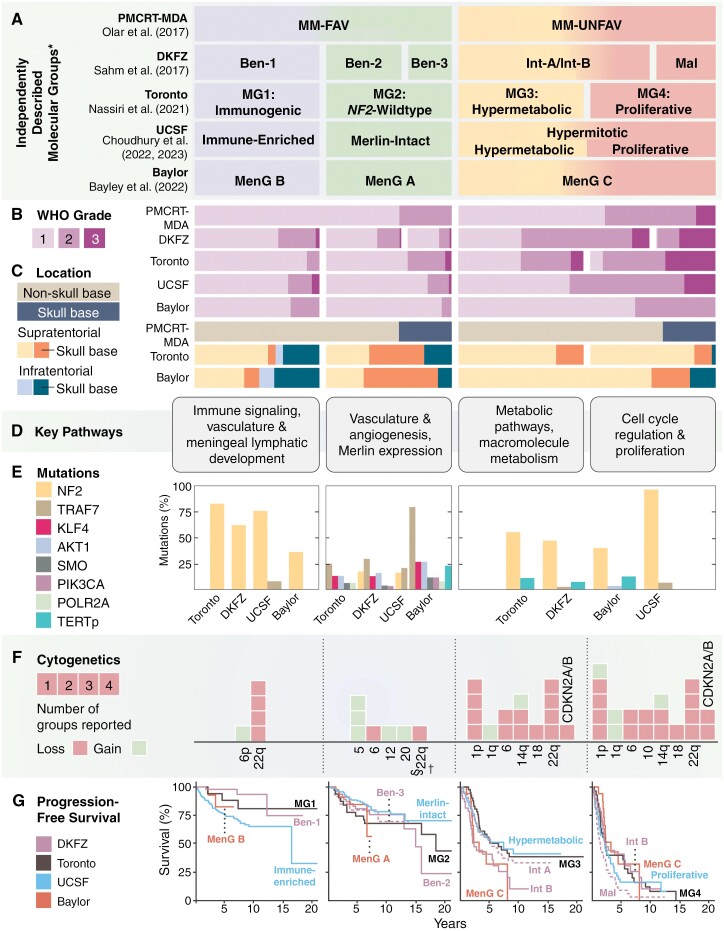
(A) Different meningioma molecular/methylation classifications discovered by independent groups arranged based on approximately how they correlate with one another based on common biology, alterations, and outcome (read from top to bottom). (B) Relative distribution of meningiomas belonging to each WHO grade in each molecular or methylation group. (C) Relative proportion of meningiomas based on location in either a skull base or non-skull base location in the supratentorial or infratentorial compartment in datasets where tumor location was available. (D) Key transcriptomic pathways found to be overexpressed in meningiomas belonging to each molecular or methylation group, grouped into 4 main sets of pathways. (E) Relative distribution of common meningioma driver mutations found in cases with more benign biology (left) and more biologically aggressive cases (right). (F) Proportion of different chromosomal alterations seen in each molecular or methylation group. (G) progression-free survival (PFS) of meningiomas belonging to each recently published molecular or methylation group based on the original publication’s cohort. *Importantly to note, these groups may not correlate with one another precisely on a one-to-one basis and as a result, the PFS curves of different groups may be repeated in different panels. ^†^For instance, while many meningiomas from the Ben-3 methylation subclass share commonalities with Merlin-intact or NF2-wild-type cases (eg, absence of 22q deletions, presence of chromosome 5 gain, angiomatous histology), some cases may classify into other molecular groups eg, immunogenic or hypermetabolic groups. Similarly, some cases of Ben-3 do have 22q deletions as well. Int-A and Int-B meningiomas may not precisely separate into hypermetabolic and proliferative cases. PMCRT, Princess Margaret Cancer Research Tower; DKFZ, German Cancer Research Center; UCSF, University of California San Francisco; MM-FAV, meningioma methylation group favorable; MM-UNFAV, meningioma methylation group unfavorable; Ben, benign; Int, intermediate; Mal, malignant; MG, Molecular Group; MenG, Meningioma Group; NF2, neurofibromatosis 2; TRAF7, Tumor necrosis factor receptor-associated factor 7; KLF4, Krüppel-like factor 4; AKT1, RAC(Rho family)-alpha serine/threonine-protein kinase; SMO, Smoothened; PIK3CA, phosphatidylinositol-4,5-bisphosphate 3-kinase, catalytic subunit alpha; DNA-directed RNA polymerase II subunit RPB1; TERTp, Telomerase reverse transcriptase promoter; CDKN2A/B, cyclin-dependent kinase inhibitor 2A/B.

Subsequently, the integration of genome-wide DNA methylation, mRNA expression, and copy number alterations resulted in the discovery of 4 stable molecular groups (MGs) of meningioma (Figure 3).^64^ Classification by MG was found to have improved prognostication potential and biological relevancy compared to WHO grade and classification using any single epigenomic or genomic platform alone. MG1 or “immunogenic” meningiomas were defined as *NF2*-mutant, copy-number neutral cases enriched in immune-related transcriptomic pathways. MG2 meningiomas were found to be enriched for non-*NF2* mutations and angiogenic processes, earning the “*NF2*-wild type” designation. MG3 and MG4 meningiomas were enriched for prognostically unfavorable alterations including *TERT*p mutation and homozygous loss of *CDKN2A/B*, in addition to novel somatic mutations in *KDM6A*, *CHD2*, and *PTEN*, and these tumors had a significantly higher degree of chromosomal instability. On transcriptomic analysis, several metabolic pathways including those involved in nucleotide and lipid metabolism were upregulated in MG3 meningiomas, giving this group its “hypermetabolic” name. MG4 or “proliferative” meningiomas were found to be enriched for cell cycling pathways including *MYC*, *FOXM1*, and *E2F* pathways, had the highest mutational and copy number burden, and were associated with the worst clinical outcomes.^64,112–116^

Around the same period of time, other molecular classification schemes were discovered by others in independent cohorts. Choudhury et al. uncovered 3 stable methylation groups with unique clinical outcomes and biology: Merlin-intact (MI), immune-enriched (IE), and hypermitotic (HM). MI meningiomas, analogous to MG2 cases (NF2-wild type), were largely benign tumors enriched for non-*NF2* mutations such as *TRAF7*, *AKT1*, and *KLF4*. IE meningiomas, similar to MG1 (Immunogenic), were found to have significant immune cell infiltration and increased expression of *HLA* and meningeal lymphatic genes including *LYVE1, CCL21,* and *CD3E*. HM meningiomas were clinically aggressive cases with poor outcomes enriched for *FOXM1* cell proliferation pathways.^37^ Subsequent reanalysis of the HM group revealed 2 distinct subgroups within it: one subgroup enriched in pathways related to macromolecule metabolism (resembling the MG3 Hypermetabolic meningiomas with intermediate to poor outcomes) and the other enriched for cell cycle pathways that had the worst clinical outcomes (similar to the MG4 Proliferative meningiomas). These findings seem to support the concept of either 4 distinct MGs matching those discovered by Nassiri et al. or 3 epigenetic groups with one group that could be further split into 2 subgroups with distinct clinical outcomes and gene expression signatures.^117^ Bayley et al. also found 3 methylation groups of meningioma based on integration of DNA methylation, RNA expression, *NF2* status, and degree of chromosomal instability in a cohort of primary CNS WHO grade 1 and 2 meningiomas. By their classification, MenG A meningiomas were almost entirely CNS WHO grade 1, had no cytogenetic changes, and were *NF2*-wild type, corresponding to the MG2 and MI groups described above. MenG B meningiomas were all *NF2*-deficient, had a low degree of chromosomal instability, and had overall good clinical outcomes, seemingly matching the MG1 and IE groups. MenG C meningiomas were NF2-deficient, had a high burden of copy number alterations including 1p loss, and like the MG3, MG4, and HM groups, have the worst clinical outcomes.^118^ Each of these molecular classification systems tends to complement and/or outperform contemporary WHO grading alone in predicting clinical outcomes. Despite differences in nomenclature and classification, which may be attributed to the use of different epigenomic/genomic platforms and bioinformatic methods utilized in these separate studies, these molecular classifications share a meaningful degree of common biology, particularly when considering they were discovered in completely non-overlapping, independent cohorts (Figure 3; Supplementary Table 1). These studies together have not only demonstrated the value of utilizing orthogonal bioinformatic methods to independently produce stable molecular/methyation groups, but have also generated a wealth of genomic/epigenomic data as a valuable resource for future studies. An important caveat is that these classifiers may be insufficiently powered to include rare subsets of poor-performing *NF2*-wild-type tumors including meningiomas with *BAP1* mutations, and management of these unusual, but clinically important cases should be carefully considered on an individual patient basis. Upcoming efforts, including those by the cIMPACT-NOW group, will focus on reconciling the nomenclature of these different molecular classifications to reach a consensus that can be implemented into a future unified grading system.

One of the additional challenges hindering the routine implementation of these molecular classifications is the requirement for sequencing and/or methylation array technology that may not be accessible at all centers. This is in addition to other barriers to genomic testing that include but are not limited to: financial reimbursement, site-dependent experience in data analysis and interpretation, and uncertainty in selecting the specific assays or tests to perform. One method of addressing these challenges may be with the use of proteomics to identify immunohistochemical (IHC) markers enriched in each molecular group or specific combination of markers such that tumors may be molecularly subtyped in the future without genomic data at all. For this to be clinically validated, IHC stains will need to be multiplexed in large, molecularly annotated meningioma cohorts, ideally in a prospective manner, and analyzed by experienced neuropathologists blinded to molecular classification and each other’s annotations.

Additional uncertainty may arise in deciding on which molecular classification or the above referenced integrated grading system to use. While there are notable differences in classification or prognostication in models that are trained on clinical endpoints (eg, integrated molecular-morphological meningioma classification or integrated WHO grade) versus the unbiased molecular group classifications detailed above, these methods all provide some degree of additive prognostic information to traditional grading and for the time being, may be utilized interchangeably based on the available resources of each institution.^64,92,93,117–119^ Efforts to expand access to genomic and methylation testing for meningiomas will not only aid in prognostication, but help ensure continued progress in better understanding the biology of these tumors. To this point, while the DNA methylation and gene expression patterns of some meningiomas appear to remain stable between the primary and recurrent case, the effect of accumulating epigenetic and genomic alterations including progressive chromosomal instability with multiply recurrent cases (including cases that were completely resected at one point), metastatic meningiomas, and cases following receipt of radiotherapy (RT) still need to be further investigated.^109,120,121^

An emerging area of interest for meningiomas is the use of liquid biopsy for diagnosis and subtyping. The use of cell-free methylated DNA immunoprecipitation and high throughput sequencing (cfMeDIP) on patient plasma was able to effectively differentiate meningiomas from other radiographic mimickers such as solitary fibrous tumors, dural-based metastases, and chordomas.^122,123^ Extracellular vesicles from the plasma of meningioma patients quantitatively correlated with the extent of resection and their contents were found to reliably recapitulate the methylation signatures of the parent tumor, including copy number and mutational profile.^124^ Additional work has found that plasma-based DNA methylation signatures of meningioma patients may have similar prognostic potential as in the tumor tissue for differentiating between high- and low-risk cases. These findings collectively need to be further validated in larger, external validation cohorts with matched tissue profiling before clinical translation may be feasible.^125^

## Diagnosis and Imaging

Many meningiomas are diagnosed when patients become symptomatic from either mass effects or seizures.^126–128^ On non-contrast computed tomography (CT), up to 25% of meningiomas will have some degree of calcification, which may be sometimes associated with slower tumor growth and lower WHO grade.^129^ Magnetic resonance imaging (MRI) is the preferred modality for confirming the radiographic diagnosis with most meningiomas being isodense to cortex on all sequences, and approximately 50% may be associated with some perilesional edema.^130^ Secretory, microcystic, angiomatous, and lymphoplasmacyte-rich meningiomas are histologic subtypes known to cause a disproportionately large degree of edema relative to tumor size and may portend increased risk of postoperative complications.^131,132^ Almost all meningiomas avidly enhance with gadolinium contrast and up to 72% have a dural tail.^133^ Whether the dural tail consistently contains neoplastic meningioma cells requiring treatment or simply represents reactive or inflammatory dural thickening is controversial.^134–138^ Vascular imaging, often CT- or MR-angiogram (CTA, MRA) and/or CT/MR-venogram (CTV, MRV) can help assess the involvement of nearby vascular structures for treatment planning, which is particularly important around the skull base or dural venous sinuses. Formal cerebral angiography is more rarely performed but may be indicated if noninvasive vascular imaging provides insufficient information or if preoperative embolization is planned. There remains controversy around whether preoperative embolization reliably leads to decreased blood loss intraoperatively and its use may be associated with an increased risk of postoperative venous thromboembolism.^139,140^ Therefore, preoperative embolization is not a recommended strategy for all meningiomas and decisions surrounding its use must be made on a case-by-case basis.

There are currently no standardized response criteria or clinical trial endpoints for meningioma studies. Previous trials have used a modification of the Macdonald criteria (initially developed for high-grade gliomas), the Response Assessment in Neuro-Oncology (RANO) criteria for high-grade gliomas, or the Response Evaluation Criteria in Solid Tumors (RECIST) criteria for systemic cancers.^141–146^ While some trials have used a reduction in lesion size as a radiographic endpoint, meningioma control is better encapsulated by lack of growth (size stabilization) as a decrease in size occurs in only a relative minority of cases treated with RT over time (approximately 20%–30% of cases).^147–149^ Additionally, while overall survival is often the gold standard for determining treatment efficacy, the long follow-up time required to reach this endpoint for all but the most aggressive meningiomas presents a significant challenge, particularly for clinical trials. The RANO Working Group instead proposed that 6-month PFS could be a viable endpoint for meningioma drug trials with a 25% increase in the tumor’s bidimensional product representing definitive progression.^141^ For patients enrolling in clinical trials, collection of pretreatment MRIs will be important to confirm adequate progression of the tumor during trial follow-up. In the future, measurement of tumor volume and assessing changes in the rate of tumor growth before and after treatment may be another method of evaluating the efficacy of novel therapies.^150^ For reporting in retrospective studies, the ICOM proposed the definition of tumor progression to be any radiographic progression that leads to a change in the clinical management of the tumor (eg, from observation to consideration for surgery, RT, or stereotactic radiosurgery [SRS]), thereby excluding cases of minimal radiographic growth or small volume increases followed by a plateau of stability that may not be clinically significant.^151^

**Figure 4.**
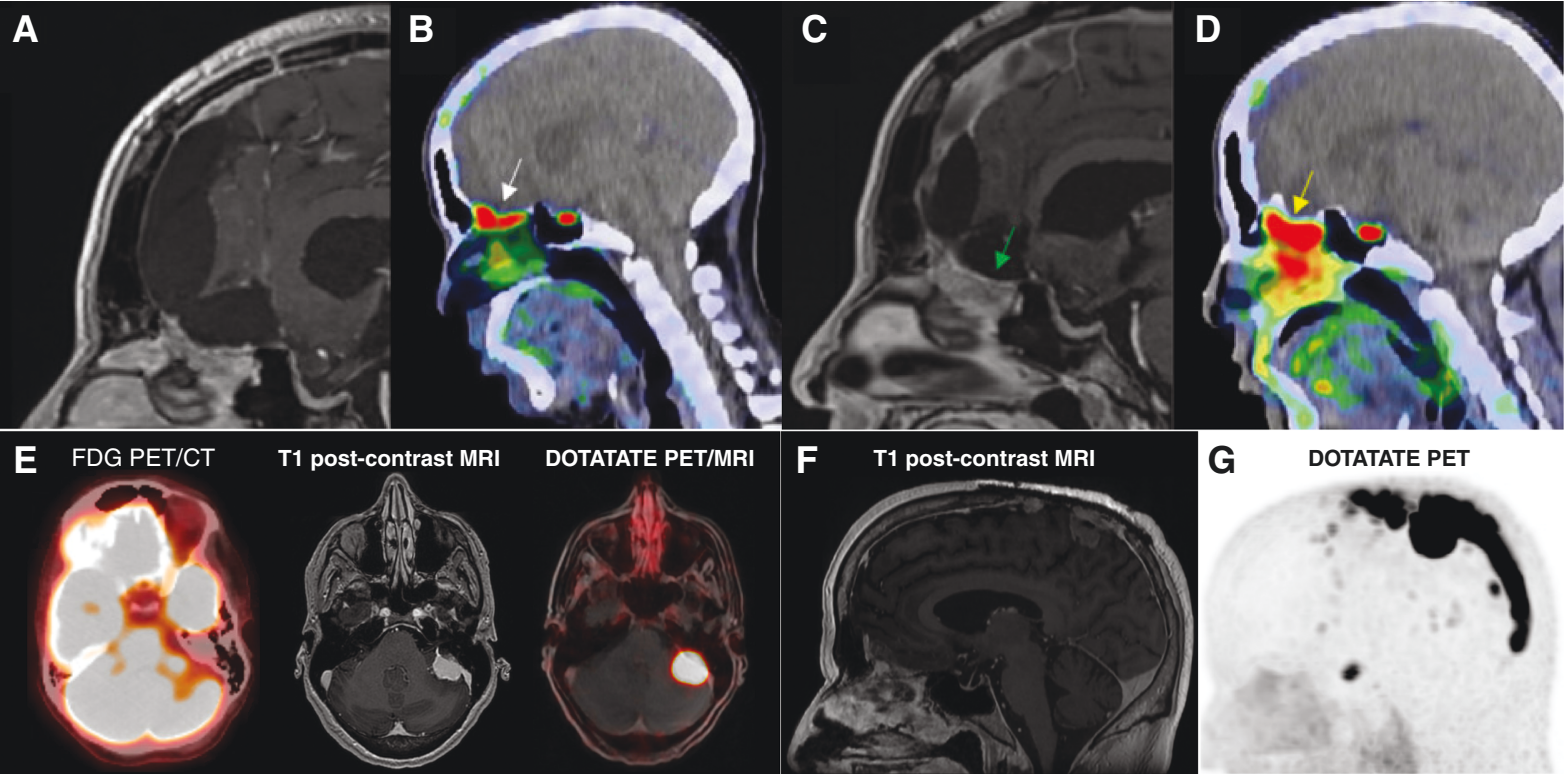
(A) Postoperative magnetic resonance imaging suggesting a gross total resection with contrast-enhancing reactive changes only. (B) Positron emission tomography (PET) imaging showing focal uptake along the cribriform plate (standardized uptake value 7.43, white arrow) suspicious for residual disease. (C) Follow-up MRI 2 years later after patient declined to pursue recommended adjuvant RT with increased enhancing soft tissue signal (arrow). (D) Increased focal PET uptake in the cribriform plate suggests progression of residual disease (standardized uptake value 8.96, yellow arrow). (E) Axial brain MRI of a different patient: a 54-year-old woman with newly diagnosed breast cancer metastatic to axillary lymph nodes who was noted to have asymmetric photopenia in the left cerebellum on a staging fluorodeoxyglucose (FDG)-position emission tomography (PET) and computer tomography (CT) scan (left). T1 post-contrast brain magnetic resonance imaging (MRI) showed a multilobulated, homogeneously enhancing extra-axial mass adjacent to the left petrous temporal bone with associated edema and mass effect in the left cerebellum and cerebellar peduncle (middle). Leading differential diagnoses included a distant metastasis or a meningioma. DOTATATE PET/MRI showed markedly avid uptake in the intracranial mass (right), but not in the right breast or ipsilateral lymph nodes (not shown). A diagnosis of synchronous meningioma and locoregionally advanced breast cancer was made. The meningioma was treated with stereotactic radiosurgery (SRS). The patient underwent lumpectomy, sentinel lymph node biopsy, and adjuvant whole breast radiotherapy. At 24 months after meningioma treatment and 13 months after breast cancer treatment, the patient had no evidence of disease. (F) Sagittal T1 post-contrast brain MRI (left) and DOTATATE PET (right) of a 61-year-old male with recurrent atypical meningioma, CNS WHO grade 2, status post resection and stereotactic radiosurgery 8 years before developing multiple vertex recurrences that were treated with subtotal resection. Planning DOTATATE PET imaging revealed extensive tumor infiltration of the sagittal sinus from the vertex to the torcula. Part of this figure was originally published in The International Journal of Radiation Oncology, Biology, Physics. Prasad et al. ^68^Ga-DOTATATE PET: The Future of Meningioma Treatment (2022).^152^ FDG, fluorodeoxyglucose; MRI, magnetic resonance imaging; PET, positron emission tomography; used with permission.

An emerging imaging tool for meningiomas is positron emission tomography (PET) using somatostatin receptor (SSTR) ligands such as Gallium-68-labeled DOTATATE given that nearly all meningiomas express SSTR1/2 (Figure 4).^152^ Recently published guidelines from the RANO Working Group suggest that [^68^Ga]Ga-DOTATATE PET can be used for diagnosis, surgical resection, and RT treatment volume planning, as well as post-treatment surveillance (Figure 4).^153,154^ When compared to conventional MRI, [^68^Ga]Ga-DOTATOC PET had improved sensitivity for detecting meningiomas, particularly in areas of tumor-invading bone, locations obscured by calcifications or radiographic abnormalities, tumors centered at the skull base, or those located next to the falx.^155^ The ability to image the entire body is also advantageous for detecting systemic metastases in multiply recurrent higher grade or malignant meningiomas that although rare, will dramatically influence patient prognosis and treatment planning if identified. When correlated with SSTR2 immunohistochemistry and tumor histology, [^68^Ga]Ga-DOTATATE PET was found to be capable of differentiating between meningioma and tumor-free tissue with high accuracy, suggesting that it can be reliably used to demarcate tumor-invaded bone that may require additional drilling to maximize extent resection (particularly in the skull base; Figure 4) and also inform adjustments in RT planning in addition to response assessment after RT.^152,156–160^ Postoperatively, PET imaging may also better define residual tumor more accurately than traditional MRI and may also differentiate true tumor progression/recurrence from treatment effect.^153,161,162^ Recently, Fluorine-18-labeled SSTR-tracers such as [^18^F]SiTATE have been developed which demonstrate similarly high uptake in meningiomas while boasting lower radiation exposure and less logistic constraints for transport and clinical use compared to [^68^Ga]Ga-DOTATATE PET given its longer half-life (110 vs 68 minutes).^163,164^ Although PET imaging is a promising addition to the armamentarium for meningioma diagnosis and treatment, its limitations include the still sparse data on cost-effectiveness, physiologic uptake near certain anatomic structures such as the pituitary gland, and tracer uptake by other tumors or non-neoplastic diseases that may also express SSTR.^153,165^ Furthermore, additional prospective work and multicentre clinical trials are needed to link these positive findings from often single-institution retrospective studies with demonstrable improvements in clinical outcomes.^152^

While many meningiomas are diagnosed symptomatically, approximately 20% are found incidentally, a proportion likely to increase with an aging population with increased access to neuroimaging.^128,166^ Incidental meningiomas can be a source of significant anxiety for patients, an economic burden due to the need for regular follow-up imaging, and a clinical dilemma for clinicians due to their unpredictable biology given the absence of diagnostic tissue.^128,166–168^ Natural history studies on incidental meningiomas typically only extend to the 10-year follow-up mark and most have found a relatively slow rate of growth (average < 5% volumetric increase per year). Approximately 5–8% of patients will develop new symptoms during a mean follow-up period of 4.1 years (standard deviation 2.4 years).^167^ Imaging features that may portend a higher risk of progression of an incidental meningioma include: lack of calcification, hyperintensity on T2-weighted MRI, presence of peritumoral edema, large tumor volume at diagnosis (>10 cm^3^), non-skull base location, and closer proximity to a dural venous sinus.^39,114,167,169–175^ There are currently no standardized guidelines for the interval or duration of monitoring for incidental meningiomas. Although most meningiomas that progress will do so within 5 years of observation, some cases can remain indolent for a longer period before demonstrating accelerated recurrence or growth. Consequently, many clinicians may follow incidental meningiomas in younger patients for a longer duration of time, progressively lengthening the interval between neuroimaging while elderly patients may be discharged from follow-up earlier after a confirmatory period of radiographic stability.^128,167^ Several prognostic models such as IMPACT (Incidental Meningioma: Prognostic Analysis Using Patient Comorbidity and MRI Tests) have been developed to assist clinicians in tailoring follow-up to a specific patient based on individualized clinical and tumor factors but these models all require prospective validation.^128,166,176–178^ Upfront treatment of incidental meningiomas is also an option, with surgical resection for often larger tumors, and SRS as a reasonable option for smaller volume cases or for patients with contraindications to surgery.^179–181^ Decisions to treat usually hinges on a combination of patient wishes, clinician preference, and tumor factors including proximity of the meningioma to critical neurovascular structures such that further enlargement or growth could make later resection more challenging or higher risk. Newer technologies such as liquid biopsy or ^18^F-FLT PET could be used to help predict the risk of recurrence non-invasively and better individualize management for these cases.^125,182^ While SRS improves radiographic local control of asymptomatic meningiomas compared to observation, this may not translate to a reduced risk of developing new symptoms over time.^181^ Furthermore, even though a subset of incidental meningiomas will grow radiographically, these changes may not become clinically significant until tumor size reaches a certain threshold or nears eloquent brain areas. As usual, treatment decisions should weigh the risks of progression versus the risks of intervention, while also taking into consideration the psychosocial, neurocognitive, and socioeconomic effects of active surveillance for the patient versus upfront treatment.^183,184^

## Surgical Management

**Figure 5.**
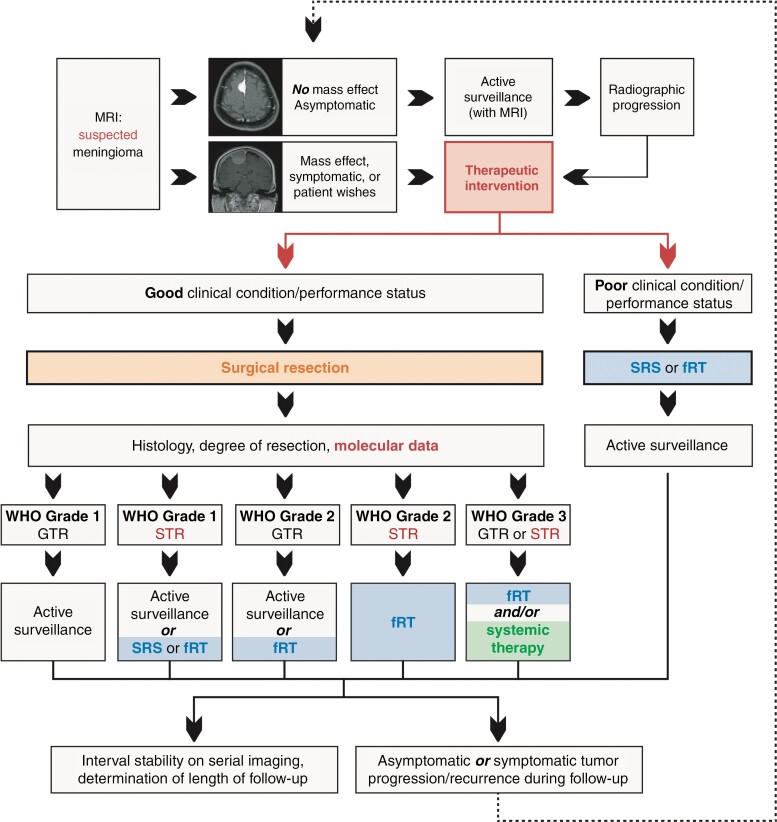
Summary of most contemporary treatment guidelines for the management of meningiomas based on WHO grade, extent of resection, with the incorporation of molecular data if available. Content for this figure was partly adopted from Goldbrunner et al. EANO Guideline on The Diagnosis and Treatment of Meningiomas (2021) published in Neuro-Oncology.^127^ Used with permission. MRI, magnetic resonance imaging; SRS, stereotactic radiosurgery; fRT, fractionated external beam radiotherapy; GTR, gross total resection; STR, subtotal resection.

Surgery remains the mainstay of treatment for growing or symptomatic meningiomas (Figure 5). Goals of surgery, as defined by the 2021 EANO guidelines, are predominantly to obtain a tissue diagnosis, relieve mass effect, and alleviate neurologic symptoms if present.^127^ Notably, extent of resection is an important correlate of outcome, and maximal safe resection should be sought while minimizing neurologic morbidity in all symptomatic cases. To this end, surgical adjuncts including neuronavigation, ultrasonography, and intraoperative neuromonitoring are critical for tumors located in highly eloquent areas such as the cerebellopontine angle or foramen magnum, to reduce the risk of incurring permanent neurologic injury. Since some meningiomas are intimately associated with critical neurovascular structures, complete resection without unacceptable morbidity is not always possible; it is, therefore, important to standardize a maximally beneficial degree of resection for these cases in a meaningful way.

The Simpson grade, first introduced in 1957, describes the surgeons’ assessment of the extent of resection for meningioma. It ranges from Simpson grade 1 (complete resection of tumor, affected dural attachment, and bone) to 5 (decompression/biopsy only) with higher grades associated with higher rates of recurrence.^185–188^ Complete tumor resection may be designated as Simpson grades 1, 2, or 3 depending on whether the underlying dura is resected, coagulated in situ, or left intact, respectively. While the Simpson grade has historically been a major predictor of postoperative PFS, its role in modern meningioma surgery has become somewhat controversial.^161,186,187,189–191^ For example, recent studies have shown that resecting the underlying dura (Simpson grade 1 resection) may not be associated with improved outcomes compared to other Simpson grades.^190,191^ This is important in cases of meningiomas originating from the skull base, where aggressive dural resection may be associated with increased risk of complications such as CSF leak or for meningiomas involving dural venous sinuses where hemorrhage, venous infarct, or air embolism are notable risks when pursuing aggressive resection.^186,190^ In these cases, achieving maximal tumor resection without excising the underlying dura may decrease morbidity without meaningfully affecting PFS. Additionally, skull base meningiomas are more likely to exhibit more benign biology, which is used as evidence for opposing viewpoints. On one hand, striving for a Simpson grade 1 resection in these complex cases may confer unnecessary surgical risk, thereby supporting a more conservative philosophy. On the other hand, complete resection in the context of a meningioma with more benign biology may provide an opportunity for robust oncologic cure, obviating the need for further surgery or adjuvant RT and this view supports a more aggressive surgical approach.^192–194^ The optimal strategy in these cases will depend largely on the surgeon’s comfort level, experience, and of course, the patient’s wishes and their risk tolerance for neurologic deficits, temporary or permanent, that may be incurred in an effort to achieve a potential cure. As an additive step to a Simpson grade 1 excision, a “Simpson grade 0” resection, whereby an additional 2-cm margin of surrounding dura is removed, has been proposed primarily for convexity-located meningiomas where this is most feasible.^195^ However, there are currently no well-established guidelines for the extent of dural resection recommended to optimally prolong time to recurrence and adjunctive technologies such as Raman spectroscopy or SSTR PET may help to better define this moving forward.^152,156,161,196–198^

Considering these limitations in Simpson grading, there has been movement towards defining extent of resection as simply either gross total resection (GTR), indicating cases where all tumor is removed regardless of how the underlying dura is handled (analogous to Simpson grades 1–3), and subtotal resection (STR), indicating cases where a portion of gross tumor is left behind (Simpson grades 4/5). This definition has been adopted by organizations such as the European Organization for Research and Treatment of Cancer (EORTC) and the Radiation Therapy Oncology Group (RTOG).^127^ However, the role of either Simpson grading or extent of resection in the context of meningioma molecular classifications has yet to be adequately explored.

The different surgical approaches to intracranial meningiomas are vast and a comprehensive review of each approach is beyond the scope of this article. The latest evolution in surgical techniques for meningiomas emerged with improvements in endoscopic technologies, permitting expanded endonasal approaches (EEA) to the anterior skull base including olfactory groove and tuberculum sella meningiomas (or, less commonly, tumors in the middle fossa, posterior fossa, or orbit) for appropriately selected patients. Tuberculum sellae meningiomas are the prototypical candidates for endoscopic resection through an EEA and a trend towards better visual outcomes at the cost of higher CSF leak rates for these patients has been found when compared to open, transcranial approaches.^199–201^ Tuberculum sellae meningiomas selected for EEA often tend to be smaller in size with less perilesional edema and no vascular encasement (which is a contraindication for most surgeons using an endoscopic approach).^201^ Overall, there is insufficient evidence demonstrating the universal superiority of one surgical approach over another, and each case should be individualized based on patient and tumor factors in addition to the surgeon’s comfort level and expertise.^127^

## External Beam Radiotherapy for Meningiomas

In addition to surgical resection, RT is the only widely accepted treatment modality for meningiomas. RT may be prescribed as primary treatment or as an adjunct to surgery, either immediately following surgery as adjuvant therapy or delayed as salvage treatment at the time of tumor progression/recurrence. The optimal timing of adjuvant RT is currently unknown. The recent EANO guidelines suggest primary fractionated RT as a treatment option for symptomatic patients or those with sufficiently large meningiomas beyond the treatment limits of stereotactic radiosurgery (SRS) who cannot undergo surgery due to underlying comorbidities, unacceptably high surgical risk, or patient preference. These same guidelines also recommend RT as an adjunct to surgery in all patients with CNS WHO grade 3 meningiomas or CNS WHO grade 2 cases following subtotal resection. Recent evidence has suggested that RT may have a role even for patients with CNS WHO grade 1 meningiomas that cannot be completely resected, a cohort that had worse PFS than completely resected and irradiated CNS WHO grade 2 meningiomas in the non-randomized RTOG-0539 phase II clinical trial (Figure 5).^127,202^

The use of adjuvant RT in all CNS WHO grade 3 meningiomas and partially resected CNS WHO grade 2 meningiomas (so-called “high-risk” cases) is supported by the same RTOG-0539 trial, which treated these cases with intensity-modulated RT (IMRT) with 60 Gy over 30 fractions.^203^ This achieved a 3-year PFS of 58.8% and overall survival of 78.6% in 51 enrolled patients, with minimal adverse effects (one grade 5 necrosis-related complication in a patient with a large RT treatment field, all others being grades 1–3 adverse events). Additionally, EORTC 22042-26042, a non-randomized phase II study of patients with WHO grade 2 meningioma who underwent complete resection and postoperative RT (60 Gy), achieved an encouraging 3-year PFS of 88.7%.^204^ With improvements in RT technology, dose escalation has been proposed as a strategy for higher-grade (WHO grade 2 or 3) meningiomas. The phase II MARCIE trial utilized a carbon-ion (C12) boost of 18 Gy over 6 fractions combined with IMRT or fractionated stereotactic RT of 50.4 Gy/28 fractions for incompletely resected WHO grade 2 meningiomas, with resultant 3-year PFS and local control rates of 80.3% and 86.7%, respectively. However, a higher-than-expected proportion of patients developed radiation-induced contrast enhancement post-treatment and the study was prematurely terminated due to one treatment-associated death.^205^ A large, single-center retrospective study from Toronto found that dose escalation of conventional photon-based RT to 66–70 Gy over 33–35 fractions for WHO grades 2 and 3 meningiomas (both as adjuvant and salvage treatment) led to improvements in local control and PFS compared to standard dose RT regimens (59.4–60 Gy/30–33 Fr), without a significant difference in treatment-related adverse events, although the authors acknowledged likely underreporting of these toxicities.^206^ Given these uncertainties, a randomized controlled trial may be needed to answer the question of optimal RT dosing for higher-grade meningiomas. Several other retrospective studies also supported the use of adjuvant RT in CNS WHO grade 2 and 3 meningiomas but these studies were often limited by small sample sizes, non-standardized RT doses/techniques, lack of distinction between local and out-of-field treatment failures, and evolving WHO criteria.^207,208^ There continues to be controversy surrounding the benefit of adjuvant RT in patients with completely resected CNS WHO grade 2 meningiomas, a group wherein the guidelines remain equivocal. This critical question is being addressed with the ongoing phase III randomized trials NRG BN-003 (NCT03180268) and ROAM/EORTC-1308 (ISRCTN71502099) with results of both trials pending.^209–212^

The conventional use of WHO grading to stratify meningiomas into different treatment arms should also be considered. The WHO criteria for CNS WHO grade 2 and 3 meningiomas (the cases that are most often selected for adjuvant RT clinically) have undergone several updates of from 2000 to 2021.^3,213–215^ Clinical trials that accrue over several years may require central pathological review and regrading or be limited by this confounder. Furthermore, apart from the most recent 2021 classification, all previous WHO grading systems were entirely based on histopathology and in some instances may be susceptible to differences in interpretation between pathologists.^216,217^ In this emergent molecular era of meningioma classification, the WHO grade has been shown to be less predictive of outcome than nearly all molecular classification systems although robust, large-scale validation of these classifications are still needed, particularly as it pertains to response to RT. Despite the associated challenges, it will be important to consider prognostic molecular alterations when it comes to future selection of patients for adjuvant RT. When DNA methylation was performed on 38 CNS WHO grades 2 and 3 meningiomas from the phase II EORTC 22042–26042 clinical trial that received different degrees of surgical resection, loss of chromosome 1p and unfavorable DKFZ methylation class were found to be associated with worse 3-year PFS, although statistical significance was not met.^218^ Recently, a 34-gene expression signature was developed that appeared to outperform WHO grade and several other molecular prognostic systems in accurately predicting 5-year PFS. Using this prognostic signature, meningiomas were able to be stratified into cases at high- and low-risk of recurrence following surgery.^219^ Although this gene expression biomarker was robustly validated in large external cohorts where postoperative management for up to 29.8% of cases could be refined, these cohorts spanned multiple decades of time and included only 210 patients who actually received postoperative RT. Therefore, further validation is needed to translate this signature to specifically RT-treated meningioma cases before its utility for determining response to RT can be definitively established.^220^

Finally, patients undergoing primary RT for meningioma in lieu of surgery may undergo either SRS or fractionated external beam RT. While both have been associated with high rates of tumor control, the latter may be preferred for larger tumors (typically larger than 2–3 cm in maximum diameter but may be institution-dependent) or those close to radiation-sensitive structures such as the brainstem or optic nerves since fractionation optimizes normal tissue tolerance.^221–224^ Nevertheless, recent non-randomized evidence suggests that larger meningiomas may have worse outcomes with fractionated RT.^223,225,226^ Small cavernous sinus meningiomas and optic nerve sheath meningiomas; however, tend to be well controlled with primary fractionated RT and have similarly high rates of symptomatic improvement after treatment.^223,227,228^

## Stereotactic Radiosurgery for Meningiomas

SRS is defined as treatment with a single fraction of radiation, typically using doses ranging from 12–18 Gy to the 50% isodose line for Gamma Knife, 60%–70% isodose line for CyberKnife, or up to 80% for other linear accelerator (LINAC) based methods. Delivery of SRS in multiple fractions using frameless image-guided SRS systems, termed hypofractionated stereotactic radiotherapy (HSRT), has also been implemented and typically applies a dose per fraction of ≥5 Gy not exceeding 5 fractions. The multicenter retrospective IMPASSE study (*I*ncidental *M*eningioma *P*rogression During *A*ctive *S*urveillance or After *S*t*e*reotactic Radiosurgery) on small asymptomatic/incidental meningiomas demonstrated that in a large cohort matched for patient age, tumor volume, location, and imaging follow-up, meningiomas that received SRS had a tumor control rate of 99.4% compared to 62.1% in the observation arm. This suggests that SRS likely does change the natural history of some meningiomas, with the caveat that most incidental and asymptomatic meningiomas do not demonstrate clinically significant growth on long-term follow-up and can be safely observed without any treatment.^128,167,177^ Treatment may be warranted in meningiomas that are adjacent to critical structures where growth may lead to neurologic deficits or higher risk of subsequent intervention, particularly in younger patients, although this decision too must be balanced against long-term RT-associated adverse events such as cognitive decline.^208^

In a meta-analysis of non-cavernous sinus CNS WHO grade 1 meningiomas treated with SRS or HSRT, local control rates ranged from 71% to 100% (median 94.2%) while PFS ranged from 55% to 97% (median 89.4%) with a median follow-up of at least 3 years.^229^ Factors associated with improved tumor control included smaller tumor volume and patient age under 65 years.^229^ Local control and PFS rates for cavernous sinus meningiomas appear to be more favorable, with 5-year PFS rates ranging from 86% to 99% and 10-year PFS rates from 69% to 97%.^229^ Factors associated with improved local control following SRS included higher marginal dose, small-to-medium sized tumors (generally < 10 cc), CNS WHO grade 1, primary SRS (vs adjuvant), treatment within 1 year of symptom onset, female sex, younger age, and less conformal plans. By contrast, tumor volume >10 cc, parasagittal/parafalcine tumor location, and venous sinus invasion were associated with worse tumor control and an increased rate of complications after SRS.^207,208,224,230–232^

The evidence on SRS for higher grade meningiomas (CNS WHO grades 2 and 3) is limited. However, tumor control in histologically confirmed higher-grade meningiomas is typically poor, with one series reporting rates as low as 50% and 17% at 2–2.5 years for WHO grade 2 and 3 meningiomas respectively.^233^ A recent multicenter study of 233 WHO grade 2 meningiomas found a similar 3-year PFS rate of 53.9% after SRS, with a 5-year PFS rate of 33.1%. When recursive partitioning analysis was performed, 2 subgroups were identified with divergent prognoses. Poor outcomes were associated with patient age over 50 years, multiple prior resections or prior RT, and treatment volume >11.5 cm^3^.^234^ There are limited data on whether higher SRS doses or hypofractionated treatment regimens are advantageous for higher grade meningiomas and existing evidence is confounded by clinical factors such as pretreatment clinical history, treatment timing, and RT field. Therefore, prospective studies are needed, particularly for cases with treatment equipoise. Importantly, as with external beam RT, given the lack of molecular stratification in the current SRS literature, future studies should focus on incorporating molecular criteria into retrospective and prospective analyses.

## SSTR-Targeted Peptide Receptor Radionuclide Therapy

Given the fact that SSTR2 ligands can be utilized for either diagnostic (eg, ^68^Ga) or therapeutic purposes (eg, ^177^Lu or Y), the concept of theranostics has gained traction in meningiomas.^235^ Several mostly single- or bi-center retrospective studies have been completed with promising results in terms of achieving stable disease in progressive, pretreated meningiomas.^236,237^ The uptake of the diagnostic tracer might be suitable as a prognostic marker for the efficacy of this therapy given its usually high sensitivity and specificity for its target.^238^ Recently, an EMA- and FDA-approved radiopharmaceutical for SSTR2-radioligand therapy became available for the treatment of neuroendocrine tumors, which like meningiomas, are characterized by high SSTR expression.^239^ A recent single-arm phase II study (NCT03971461) on the use of ^177^Lu-DOTATATE for progressive, intracranial meningiomas saw 6/14 patients achieving the PFS-6 threshold required for the study to progress to its second stage, currently open for enrollment in the United States.^240,241^ A randomized clinical trial to evaluate the efficacy of ^177^Lu-DOTATATE in recurrent meningioma is in preparation within the EORTC Brain Tumor Group network. Other radioligands are also currently being developed for similar applications.

## Systemic Therapies for Meningiomas

Classically, meningioma treatment has centered on surgical resection and RT. However, novel systemic agents have emerged as a possible option for recurrent or aggressive subtypes, all of which remain under investigation.^242^ These include tyrosine kinase inhibitors and monoclonal antibodies targeting vascular endothelial growth factor (VEGF) signaling pathways.^243–247^ A phase II trial of the multikinase inhibitor sunitinib which targets VEGF and platelet-derived growth factor receptors, among others, in CNS WHO grade 2 and 3 meningiomas showed a PFS-6 rate of 42%, meeting the primary endpoint.^243^ A phase II trial of bevacizumab (a monoclonal antibody against VEGF-A) in recurrent meningiomas reported a PFS-6 rate of 77% in grade 2 and 46% in grade 3 meningiomas, suggesting anti-tumor activity.^248^ The Alliance A071401 trial is the first genomic-driven phase II study in which patients with recurrent meningiomas are genotyped and assigned to treatment with vismodegib for tumors with *SMO* mutations, abemaciclib for cases with CDK alterations, capivasertib for tumors with *AKT* or *PI3K* mutations and a FAK inhibitor (GSK2256098) for *NF2*-mutant cases. GSK2256098 was well tolerated and demonstrated promise in achieving a PFS-6 of 83% in progressive CNS WHO grade 1 meningiomas and 33% in CNS WHO grades 2/3 cases. Cytotoxic and hormonal agents, including trabectedin, somatostatin agonists, and progesterone antagonists, have demonstrated less clinical efficacy.^247,249–257^

Immunotherapy has shown promise in treating solid organ tumors, and recently there has been growing interest in its role in meningiomas despite the challenges of their usually immunologically quiet microenvironment and low tumor mutational burden.^258^ In a single-arm, open-label phase II trial (NCT03279692), patients with progressive CNS WHO grade 2 and 3 meningiomas were treated with pembrolizumab, a PD-1 inhibitor, which met the primary endpoint and achieved a PFS-6 of 48% with a median PFS of 7.6 months.^259^ In the same trial, 20% of patients experienced one or more grade-3 or higher adverse events associated with treatment. A trial of nivolumab monotherapy in similarly progressive high-grade meningiomas failed to demonstrate improvement in PFS-6 (PFS-6 42.4%); however, 2 patients with high tumor mutational burden had increased immune cell proliferation and were long-term survivors.^260^

Thus far, more trials are needed to identify better systemic therapies for meningioma patients. Results from several published and completed clinical trials are summarized in Table 3. Given the lack of current options for treatment-refractory meningiomas, additional agents are needed. The results from ongoing trials may highlight the importance of molecular classification on patient selection for targeted therapies as opposed to stratification based on WHO grade alone. There are several ongoing clinical immunotherapy trials on the use of nivolumab, ipilimumab, and avelumab (NCT03173950, NCT04659811, and NCT03267836) for meningiomas and other CNS tumors, the results of which may yield interesting treatment insights for the future (Table 4).

**Table 3.** Selected Completed and Published Clinical Trials on Systemic Therapy in Meningiomas

Corresponding author	Year	Study title	Study population and key eligibility criteria	Total patients	Systemic/experimental agent	Primary endpoint	Outcome	+/− Primary outcome	Ref
Chamberlain	2004	Temozolomide for treatment-resistant recurrent meningioma	Progressive WHO grade 1 meningiomas and KPS ≥ 60	16	Temozolomide (75 mg/m^2^ for 42 days followed by 28-day break)	PFS-6	0%	−	^261^
Wen	2009	Phase II study of imatinib mesylate for recurrent meningiomas (North American Brain Tumor Consortium study 01–08)	Recurrent meningiomas with KPS ≥ 60	23 (13 benign (WHO grade 1), 5 atypical (WHO grade 2), 5 malignant (WHO grade 3))	Imatinib (600–800 mg PO daily)	PFS-6	29.4% (45% for benign, 0% for atypical or malignant)	−	^262^
Wen	2010	Phase II trials of erlotinib or gefitinib in patients with recurrent meningioma	Recurrent meningiomas and KPS ≥ 60	25 (16 gefitinib, 9 erlotinib)	Gefitinib (500–1000 mg PO daily), erlotinib (150 mg PO daily)	PFS-6	28% (25% gefitinib, 33% erlotinib)	−	^263^
Reardon	2011	Phase II study of Gleevec® plus hydroxyurea (HU) in adults with progressive or recurrent meningioma	Progressive/recurrent meningioma and KPS ≥ 60	21	Hydroxyurea (500 mg PO BID) and Imatinib (400–800 mg PO daily)	PFS-6	61.9% (87.5% WHO grade 1, 46.2% WHO grade 2/3)	+	^264^
Wen	2014	Phase II study of monthly pasireotide LAR (SOM230C) for recurrent or progressive meningioma	Recurrent or progressive meningioma with KPS ≥ 60	34 (18 cohort A/malignant, 16 cohort B/benign)	Pasireotide (60 mg LAR IM monthly)	PFS-6	17% cohort A, 50% cohort B	−	^253^
Kaley	2014	Phase II trial of sunitinib for recurrent and progressive atypical and anaplastic meningioma	Refractory recurrent WHO grades 2–3 meningiomas	36	Sunitinib 50 mg PO daily	PFS-6	42%	+	^243^
Raizer	2014	A phase II trial of PTK787/ZK 222584 in recurrent or progressive radiation and surgery refractory meningiomas	Surgery and radiotherapy refractory recurrent meningiomas and KPS ≥ 60	22 (14 WHO grade 2, 8 WHO grade 3)	PTK787/ZK 22585 (500 mg PO BID)	PFS-6	54.4% (64.3% WHO grade 2, 37.5% WHO grade 3)	+	^244^
Verschraegen	2015	Double-BLIND PHASE III randomized trial of the antiprogestin agent mifepristone in the treatment of unresectable Meningioma: SWOG S9005	Progressive or refractory meningioma with prior radiotherapy, PS 0–2	164 (80 mifepristone, 84 placebo)	Mifepristone (200 mg PO daily)	PFS-24	30% mifepristone, 33% placebo	−	^265^
Jensen	2016	Combined hydroxyurea and verapamil in the clinical treatment of refractory meningioma: Human and orthotopic xenograft studies	Refractory recurrent/progressive meningiomas with KPS ≥ 90	7	Hydroxyurea (20 mg/kg/day PO BID), Verapamil (120–240 mg PO daily)	PFS-6	85%	−	^266^
Graillon	2020	Everolimus and octreotide for patients with recurrent meningioma: results from the phase II CEVOREM trial	Progressive meningioma ineligible for further surgery/radiotherapy with KPS > 50	20	Everolimus (10 mg PO daily) and octreotide (30 mg LAR IM monthly)	PFS-6	55%	+	^256^
Reardon	2021	Activity of PD-1 blockade with nivolumab among patients with recurrent atypical/anaplastic meningioma: Phase II trial results	WHO grade 2 or 3 recurrent meningiomas with KPS ≥ 70	25	Nivolumab (240mg IV q2weeks)	PFS-6	42.40%	−	^260^
Preusser	2022	Trabectedin for recurrent WHO grade 2 or 3 meningioma: A randomized phase II study of the EORTC brain tumor group (EORTC-1320-BTG)	Recurrent WHO grade 2 or 3 meningiomas with PS 0–2	90 (61 trabectedin, 29 standard of care)	Trabectedin (1.5 mg/m^2^ q3weeks)	PFS-6	2.4 months Trabectedin, 4.2 months standard of care	−	^247^
Kumthekar	2022	A multi-institutional phase II trial of bevacizumab for recurrent and refractory meningioma	Progressive meningiomas with KPS ≥ 60	42 (10 WHO grade 2, 20 WHO grade 2, 12 WHO grade 3)	Bevacizumab (10 mg/kg IV q2weeks for 6 months, then 15 mg/kg IV q3weeks)	PFS-6	90% WHO grade 1, 76% WHO grade 2, 45% WHO grade 3	+	^248^
Brastianos	2022	Alliance A071401: phase II trial of focal adhesion kinase inhibition in meningiomas with somatic *NF2* mutations	WHO grade 1–3 recurrent or progressive meningiomas with NF2 mutation	36 (12 WHO grade 1, 24 WHO grade 2/3)	GSK2256098 750 mg PO BID	PFS-6	83% WHO grade 1, 33% WHO grades 2/3	+	^267^
Plotkin	2023	Prospective phase II trial of the dual mTORC1/2 inhibitor vistusertib for progressive or symptomatic meningiomas in persons with neurofibromatosis 2	NF2 patients with progressive or symptomatic meningiomas	18	Vistusertib (125 mg PO BID for 2 days per week)	Volume decrease > 20%	6% partial response, 94% stable disease	−	^268^

WHO, World Health Organization; KPS, Karnofsky performance score; PFS-6/24, progression-free survival at 6/24 months; PO, per os; IM, intramuscular; IV, intravenous; EORTC, European Organization for Research and Treatment of Cancer; NF2, neurofibromatosis-2 (NF2-SWN); BID, twice per day

**Table 4.** Selected Ongoing Clinical Trials on Systemic Therapies in Meningiomas

Principal investigator	Estimated completion	Study design	Study population and key eligibility criteria	Systemic/experimental agent	Primary endpoint	NCT #
Jiayi Huang	2023	Neoadjuvant avelumab and hypofractionated proton radiation therapy followed by surgery for recurrent radiation-refractory meningioma	WHO grade 1–3 meningioma which has failed maximal safe resection + radiation therapy	Avelumab (10 mg/kg IV q2weeks for 3 months), proton therapy (20 CGE/5 daily fractions of 4 CGE per day)	CD8+/CD4 + tumor-infiltrating lymphocytes	NCT03267836
David A. Reardon	2024	An open-label phase II study of nivolumab and ipilimumab in adult participants with progressive/recurrent meningioma	Progressive or recurrent meningiomas with KPS ≥ 70	Nivolumab (240 mg q2 weeks), Ipilimumab 1 mg/kg q3weeks)	PFS-6	NCT02648997
Priya Kumthekar	2024	Optune delivered electric field therapy and bevacizumab in treating patients with recurrent or progressive grade 2 or 3 meningioma	Progressive or recurrent meningiomas KPS ≥ 60	Bevacizumab IV dose not specified, electric field therapy using Optune daily over 18 hours	PFS-6	NCT02847559
Priscilla K. Brastianos	2024	Vismodegib, capivasertib, and abemaciclib in treating patients with progressive meningiomas	Progressive or recurrent meningiomas	Vismodegib (PO once daily), capivasertib (PO BID days 1–4, treatment q7days), abemaciclib (PO q12h), FAK inhibitor GSK2256098 (PO BID)	PFS-6	NCT02523014
Erik P. Sulman	2025	A phase II trial of 177Lu-DOTATATE for recurrent/progressive meningioma	Progressive meningioma (any grade) with KPS ≥ 60	177Lu-DOTATATE intravenously every 8 weeks up to 4 cycles	PFS-6	NCT03971461
Recursion Pharmaceuticals	2027	Efficacy and safety of REC-2282 in patients with progressive neurofibromatosis type 2 (NF2) mutated meningiomas (POPLAR-NF2)	Progressive and recurrent NF2 meningiomas	Small molecule HDAC inhibitor REC 2282 (30–60 mg PO 3 times per week, for 3 of the 4 weeks)	PFS-6	NCT05130866
Marta Penas-Prado	2027	Phase II trial of the immune checkpoint inhibitor nivolumab in patients with recurrent select rare CNS cancers	Atypical or malignant meningioma	Nivolumab (240 mg IV q2weeks for cycles 1–2, then 480 mg q4weeks for 14 additional doses)	PFS-6, CR/PR	NCT03173950
Rupesh R. Kotecha	2028	A phase II study of cabozantinib for patients with recurrent or progressive meningioma	Progressive or recurrent meningiomas with KPS ≥ 50	Cabozantinib (60 mg PO daily for 28 days)	PFS-6	NCT05425004
Nancy Ann Oberheim Bush	2028	Stereotactic radiosurgery (SRS) and immunotherapy (Pembrolizumab) for the treatment of recurrent meningioma	Recurrent WHO grade 2 or 3 meningioma	SRS (15–20 Gy/1 Fr or 25–30 Gy/5 Fr) combined with pembrolizumab (200 mg IV on day 1 to −1 of radiation then q3weeks)	PFS-12	NCT04659811

WHO, World Health Organization; NCT, National Clinical Trial; CGE, Cobalt Gray Equivalent; KPS, Karnofsky Performance Score; PFS-6/12, Progression-free survival at 6/12 months; CR, complete response; PR, partial response; CNS, central nervous system; PO, per os; BID, twice per day.

## Quality of Life for Meningioma Patients

The impact of a meningioma diagnosis on patients is often underestimated and standardized methods or tools to assess health-related quality of life (HRQoL) are still lacking. Many patients present with symptoms that can profoundly impair HRQoL including but not limited to: seizures, motor and sensory deficits, cognitive impairment, cranial neuropathies, neuropsychiatric symptoms, and systemic symptoms such as fatigue.^269,270^ Many of these symptoms may persist long after treatment, with some patients reporting considerable effects on HRQoL as long as 10 years after surgery.^271^ Therefore, even if long-term tumor control is achieved, patients may require additional targeted interventions to help them return to their premorbid quality of life. Even surveillance imaging may be associated with significant anxiety and negative effects on HRQoL.

After surgery, patients with symptomatic meningiomas often experience a significant improvement in their symptoms and improved HRQoL in the short term. However, most demonstrate persistently reduced long-term HRQoL when compared to healthy controls.^272–274^ Of note, achieving seizure control plays a significant role in improving HRQoL, whereas multiple surgical resections and the use of adjuvant RT are associated with reduced HRQoL scores, although these results may be confounded by tumor location and extent of resection. Other predictors of lower postoperative HRQoL include lower preoperative HRQoL, larger tumor size, skull base location, and the presence of peritumoral edema.^275^ Fatigue is the most common symptom that has been reported to worsen in the post-treatment period following either surgery or SRS.^275,276^ Patients who receive RT may demonstrate improvement in some domains of HRQoL in the short term but may also experience delayed and progressive cognitive decline in the long-term. Much of this data, however, is based on older RT treatment paradigms, with modern treatment plans likely to show more favorable long-term cognitive outcomes.^274,277–279^ Increased patient support resources and counseling should be directed towards patients at high risk of persisting impairments in HRQoL both before and after treatment.^276^ There is currently a critical lack of a standardized, externally validated questionnaire targeting HRQoL for meningioma patients as well as specific interventions outside of standard tumor treatments designed to improve different HRQoL domains.^274^ Correlation between the quality of life and molecular biomarkers also remain largely unexplored despite key differences in tumor behavior between molecular groups (eg, *NF2*-wild-type meningiomas may be more likely to grow in the skull base resulting in cranial nerve deficits whereas *NF2*-altered meningiomas are more common along the convexity and may be more commonly associated with seizures). Molecular subgroup-specific quality-of-life metrics may provide an opportunity for further improvements in personalized and patient-centric care.

## Seizures in Meningioma Patients

Seizures are a common presenting symptom in patients with meningioma, occurring in up to 30% of cases preoperatively.^280,281^ Risk factors associated with increased seizure risk include recurrent disease, larger tumor size, non-skull base location, higher WHO grade, presence of peritumoral edema, and receipt of postoperative RT.^4,282,283^ The presence of brain invasion and peritumoral edema are associated with neurotransmitter alterations, ionic channel changes, and blood-brain barrier disruption, all of which may contribute to the development of a cortical epileptogenic focus.^284–287^ One study found that *NF2*-mutated meningiomas had an increased risk of preoperative seizures but only when associated with atypical histology and peritumoral edema. It was additionally found that mutations in Hedgehog signaling pathway genes (*SMO, PRKAR1A, SUFU*) in CNS WHO grade 1 meningiomas were associated with increased risk of postoperative seizures.^282^ Additional work is needed to ascertain whether specific molecular alterations can independently predict perioperative seizure risk or if the postoperative course is mainly affected by the anatomic location associated with meningiomas harboring *SMO*, *PRKAR1A*, or *SUFU* mutations.

The primary treatment for meningioma-related seizures is surgical resection, ideally GTR of the tumor.^288^ For patients in whom surgery or GTR may not be feasible, or for those whose seizures persist despite surgical resection, antiepileptic medications are often used following a similar treatment protocol as prescribed for idiopathic epilepsy. There are currently no recommendations regarding the first-line antiepileptic drug of choice for these patients, although levetiracetam is the most commonly prescribed option.^288,289^ The optimal duration of perioperative antiepileptic therapy for meningioma patients with seizures is also highly variable and there are no current guidelines or level 1 evidence to support specific practices that currently are institution or provider-specific. In general, the literature supports continuing seizure medications postoperatively for 1–2 years before tapering off in the setting of ongoing seizure freedom.^288,290,291^ The use of antiepileptic medication as prophylaxis is controversial but is generally not recommended given a lack of conclusive evidence demonstrating reduction in postoperative seizure risk.^292,293^ Nevertheless, it may be considered in patients with one or more seizure risk factors such as significant peritumoral edema.^280,294^ Furthermore, results from the ongoing multicentre, randomized controlled trial STOP ‘EM (*S*urgeons *T*rial *O*f *P*rophylaxis for *E*pilepsy in seizure naive patients with *M*eningioma) in the United Kingdom on prophylactic levetiracetam for seizure naïve meningioma patients may help inform significant changes in clinical practice.^295^

## Meningiomas in Patients With NF2-Related Schwannomatosis

Pathogenic germline alterations in the *NF2* gene, whether inherited or acquired (eg, new germline variant), result in the development of the tumor predisposition syndrome NF2-SWN.^66^ NF2-SWN is a highly penetrant autosomal dominant condition with an incidence of 1 in 25 000 to 33 000.^296–298^ While classically characterized by the development of bilateral vestibular schwannomas, 48%–75% of patients with NF2-SWN will develop meningiomas at some point in their clinical course.^299^ Compared to patients with sporadic meningiomas, patients with NF2-SWN typically develop meningiomas at a younger age and are at higher risk for developing multiple meningiomas.^75,300^ Therefore patients with these phenotypes should be screened for germline *NF2* and *SMARCE1* mutations. The majority of NF2-SWN-associated meningiomas are asymptomatic and often diagnosed during the work up for NF2-SWN or over the course of routine radiographic surveillance. When present, approximately 10% of these meningiomas will grow rapidly (defined as ≥ 2 cm^3^/year by one study) while the remainder will demonstrate no or very slow growth. New meningiomas will develop in 20% of NF2-SWN patients.^301–303^ Importantly, patients with NF2-SWN who have a meningioma have been found to have a significantly increased risk of death compared to those who do not.^304^

Given their complexity, the management of patients with NF2-SWN by multidisciplinary teams at high-volume centers has been demonstrated to improve both their quality of life and life expectancy.^304^ The majority of NF2-SWN-associated meningiomas can be safely observed including those that demonstrate slow, clinically silent growth. Surgery remains the primary treatment for symptomatic or rapidly enlarging tumors, although its risks must be weighed against the anticipated risks of additional or future operative procedures that NF2-SWN patients may need to undergo for their other neoplasms.^304^ Maximizing extent of resection remains important but even moreso here must be balanced against the risk of incurring a significant neurologic deficit that may irreparably impair their quality of life or make them ineligible for other required interventions.^302^ NF2-SWN-associated meningiomas tend to be more biologically aggressive than sporadic cases (52% are WHO grades 2 or 3), although this statistic may be confounded by a relative hesitancy on the part of most surgeons to resect the presumably more benign, slow-growing tumors in these patients.^298,301^

SRS has also been proposed as a viable treatment option for enlarging meningiomas in NF2-SWN patients with 5-year local control rates generally greater than 90%. However, distant failure rates range from 27% to 51%, and studies on the topic have been limited to small institutional case series.^305–308^ Additionally, malignant transformation remains a rare but important concern with SRS in this patient population with a significantly higher risk of malignant progression in previously benign tumors from NF2-SWN patients (up to 5–6% absolute risk increase) compared to patients with irradiated sporadic disease.^309^ Furthermore, the quality of life of NF2-SWN patients may be negatively impacted by the use of RT.^278,310,311^ It is because of these considerations that RT should be used judiciously in this patient population and in many instances, are more commonly reserved for recurrent meningiomas or tumors with unacceptably high surgical risk.^298^

Given the challenges related to NF2-SWN-associated tumors, several clinical trials have been undertaken to identify better treatment options. The AZD2014 trial in NF2-SWN patients with progressive or symptomatic meningiomas (NCT02831257) used a dual mTORC1/mTORC2 inhibitor but 12 out of 18 patients withdrew due to adverse effects. The RAD001 trial (NCT01419639) was a phase II clinical trial utilizing everolimus, an mTOR inhibitor, as monotherapy for NF2-SWN patients which although appeared to slow tumor growth, did not demonstrate any significant reduction in size. The phase II CEVOREM trial (NCT02333565) combined octreotide, a somatostatin analog, with everolimus for the treatment of aggressive and otherwise treatment-refractory meningiomas, although only 4 of the 20 patients had a germline *NF2* mutation. This trial demonstrated a significant reduction in the median tumor growth rate at 3- and 6-months, with 4 patients withdrawing due to adverse effects.^256^ A retrospective review also found slowing of meningioma tumor growth with the EGFR/ErbB2 inhibitor lapatinib (NCT00973739) in 8 NF2-SWN patients with 17 tumors.^312^ The phase II INTUITT-NF2 trial (NCT04374305) utilized brigatinib, a potent anaplastic lymphoma kinase inhibitor in combination with INK-128, a dual mTORC1/2 inhibitor for NF2-SWN associated tumors, with interim results of the brigatinib arm demonstrating radiographic response of 28% in meningiomas.^313^ While some of these treatments show promise, additional prospective trials are needed before any systemic therapies are adopted into standard of care treatment guidelines. Furthermore, translational studies incorporating radiography, molecular biomarkers (including noninvasive biomarkers), histopathology, and quality of life will be critical for improving treatment paradigms for NF2-SWN patients.

## Radiation-Induced Meningiomas

Exposure to ionizing radiation is a well-known risk factor for meningioma development. While RT improves the survival of many childhood cancers, some long-term survivors are left with secondary neoplasms as a consequence of their treatment, most commonly RIMs.^314,315^ Other rarer patient populations with RIMs include those who received low-dose cranial RT for tinea capitis in the first half of the 20th century and in survivors of the atomic bombs of World War 2.^13,15,316,317^ The latency period between initial radiation exposure and the development of RIMs typically range between 10-40 years and may be inversely associated with the initial exposure dose (higher initial dose = shorter latency period).^11,13,15,318^ Given that RIMs in childhood cancer survivors may be diagnosed 40 years after their initial treatment, imaging follow-up at fixed intervals, with more frequent follow-up for those who received high-intensity treatment may be warranted.^314,315^

RIMs are biologically and clinically distinct from their sporadic counterparts and while rare (making up only 1–2% of all meningiomas), present significant clinical challenges due to their increased biological aggressiveness, multiplicity, and resistance to standard therapies. RIMs have a much higher burden of cytogenetic changes compared to sporadic meningiomas including frequent losses of chromosomes 1p (over 50% of cases), 9p, 19q, 18q, 10p, and 22q.^10,14,319^ Notably, RIMs were less frequently found to have loss of chromosome 22q or *NF2* point mutations compared to sporadic meningiomas but had more frequent *NF2* gene fusion events. These fusions are likely secondary to misrepair of RT-associated double-stranded DNA breaks and are an alternative mechanism of *NF2* disruption. Non-*NF2* recurrent mutations including in *AKT1*, *SMO*, *TRAF7*, and *KLF4* were generally not observed in RIMs.^10,41^

Standard treatment guidelines for RIMs do not currently differ from sporadic meningiomas, with surgical resection as first line therapy for symptomatic cases. When multiple RIMs are present in the same patient, surgery should target the largest and/or symptomatic tumors first. Otherwise, active surveillance remains a safe initial strategy for these tumors, with a low rate of neurologic morbidity.^320^ The role of adjuvant RT for RIMs is unclear but may be utilized in cases of subtotally resected meningiomas or those that are higher WHO grade. However, even CNS WHO grade 1 RIMs can demonstrate aggressive behavior and many of these cases are RT-resistant. SRS in select cases however appears to be safe and well tolerated for RIMs that are not amenable to surgical resection or in patients with multiple RIMs that require treatment. Overall, tumor control rates following SRS are lower for RIMs than for sporadic meningiomas, and larger treatment volume is associated with worse PFS.^321–324^

## Spinal Meningiomas

Although rarer than their intracranial counterparts (with an incidence of approximately 0.193–0.33 cases per 100 000), spinal meningiomas are the most common intradural spinal tumors, representing 25–45% of these cases.^325–327^ CNS WHO grades 2 and 3 cases are also comparatively less common in the spine.^326–328^ Spinal meningiomas also appear to differ from intracranial meningiomas on a molecular basis and are generally more biologically benign.^329–331^ A novel molecular classification was recently proposed for spinal meningiomas with 2 major subgroups: one with predominantly *NF2* mutations and the other with *AKT1* mutations (mutually exclusive to *NF2* mutations). While both subgroups were predominantly comprised of meningiomas from benign methylation subclasses, the *NF2*-mutated subgroup was associated with intermediate outcomes and were more strongly associated with female sex, thoracic spine location, and frequent tumor calcification.^109,329,330^*AKT1*-mutated spinal meningiomas had no sex predilection and were associated with meningothelial subtype, cervical spine location, and the absence of tumor calcification.^329,330^ Interestingly, there was a small subset of spinal meningiomas with a much higher degree of cytogenetic changes that did not show a clear association with either of the above subgroups and instead more closely resembled intermediate and malignant methylation subclasses of intracranial meningiomas.^330^ This suggests that as more clinically aggressive spinal meningiomas are profiled, additional molecular subgroups may be elucidated, potentially mirroring those that have been discovered for intracranial cases.

Treatment guidelines for spinal meningiomas are the same as for intracranial meningiomas, with GTR as the usual goal of surgery. In cases where a Simpson grade 1 resection (including dural resection with patch reconstruction) may not be feasible such as for tumors with a ventral origin, a Simpson grade 2 resection with extensive dural coagulation may have comparable outcomes.^327,328,332^ Careful anatomic planning of surgical corridors may avoid the need for instrumentation in many of these cases.^333^ The role of RT for spinal meningiomas is unclear, particularly given their largely benign behavior. A review of the National Cancer Database showed that only 2.5% of 10 458 patients with spinal meningiomas received RT. Older patients with higher WHO grade tumors, larger tumors, and recurrent cases were more likely to receive RT. Interestingly, this study also reported an increase in mortality risk among “borderline” (CNS WHO grade 2) and malignant (CNS WHO grade 3) tumors that received RT following surgery compared to those that did not.^334^ Further study is needed in order to fully resolve the role of adjuvant RT or primary stereotactic body RT for spinal meningiomas.^335–337^

## Pediatric Meningiomas

Unlike in adults, meningiomas are rare in the pediatric population and account for only 2.2–3.6% of all brain tumors in this group and 0.4%–2.5% of all diagnosed meningiomas.^338–342^ Also dissimilar to their adult counterparts, pediatric meningiomas affect males and females relatively equally with a greater incidence of tumors in intraventricular and spinal locations.^341–343^ There are also a higher proportion of CNS WHO grade 2 and 3 meningiomas in pediatric patients compared to adults^338,341,344^ and a larger proportion with clear cell (CNS WHO grade 2) or papillary (associated with CNS WHO grade 3) histology.^343–345^ However, grading appeared to be less predictive of overall outcome in these cases.^338,341,346^ Patients under 3-years of age or over 12 years may have worse overall survival outcomes; the former group may be associated with higher operative morbidity and mortality while the latter have meningiomas that more closely resemble adult cases.^338^ Pediatric patients with meningiomas are more likely to have NF2-SWN than adults and these cases have a much shorter time to progression and higher mortality rate compared to non-NF2-SWN cases.^338,343^ Therefore, all pediatric patients who are diagnosed with a meningioma should be screened for *NF2* mutations and other associated rare genetic conditions that may predispose them to meningioma development.^347^ Given that spinal meningiomas are also more common in pediatric patients, full craniospinal imaging at the time of diagnosis should be considered.^340,341,343,348,349^

As in adult cases, extent of resection appears to be the most important prognostic factor, with GTR conferring both a PFS and overall survival benefit.^338,347,348^ The role of adjuvant RT is controversial, with insufficient literature to assess its utility. One meta-analysis showed no clear demonstrated benefit for PFS or overall survival, though there was likely a high degree of selection bias for irradiating aggressive tumors.^338^ Clinical decisions should be made with multidisciplinary discussion on a case-by-case basis, keeping in mind that cranial irradiation is associated with significant morbidity in children. Some clinicians advocate for second-look surgery if residual tumor is detected on postoperative imaging although evidence to support this approach is not well established.^340,350–352^

Similar to adult meningiomas, *NF2* mutations and loss of chromosome 22 are the most common alterations in pediatric meningiomas, found in 47%–72% of cases.^344,349,353^ However, other classical non-*NF2* driver mutations such as *AKT1*, *SMO*, *KLF4*, and *TRAF7* have not been described in the pediatric population.^344,349,354^ Instead, a number of different *YAP1*-fusions have been described in non-*NF2* altered pediatric meningiomas (*YAP1-MAML2; YAP1-PYGO1; and YAP1-LMO1*) and have been proposed as an alternative oncogenic driver to *NF2* inactivation.^355,356^ Preclinical studies have shown that the YAP component of these gene fusions is likely the critical driver of these tumors and the *YAP1-MAML2* fusion may be targetable through pharmacologic disruption of the YAP1-TEAD interaction.^356^

The majority of clear cell meningiomas in this population expectedly harbor *SMARCE1* mutations.^47,49–51^ DNA methylation profiling largely segregates pediatric meningioma cases from adult cases and may further separate them into 3 methylation subgroups: one group comprised almost exclusively of *SMARCE1*-mutated clear cell meningiomas, one group driven by *NF2* or chromosome 22q loss, and another mixed group containing all cases with rhabdoid histology, allelic loss of chromosome 11 and rare loss of chromosome 22.^344^ The prognostic significance of these groups remain uncertain given the rarity of cases with both molecular profiling and well annotated clinical data.

## Preclinical Models of Meningioma

Historically, there has been a paucity of cell models for meningiomas due to the slow growth rates of most primary cell lines and the tendency of most cell lines to senesce after several passages. More recently, there have been renewed efforts by laboratories worldwide to optimize primary meningioma cell cultures to better study the functional impact of specific genomic alterations and create higher fidelity preclinical models.^357,358^

There are however, several well established malignant meningioma cell lines that are commercially available. One of these such lines is IOMM-Lee,established from an intraosseous CNS WHO grade 3 meningioma, and is still commonly utilized due to its ability to readily form heterotopic and orthotopic xenografts.^359–361^ Although it harbors *CDKN2A/B* loss as a hallmark of proliferation in addition to a high burden of copy number changes, it lacks the biallelic *NF2* inactivation seen in the majority of biologically aggressive meningiomas and also contains unusual chromosomal gains of 3q, 5, and 9 that are not commonly found in meningiomas. The NCH93 meningioma cell line was similarly derived from a CNS WHO grade 3 meningioma, but unlike IOMM-Lee, contains an *NF2* frameshift mutation making it a potentially more serviceable aggressive *NF2*-mutant meningioma model that also reliably forms xenografts.^362,363^ KT21-MG1 is another aggressive meningioma cell line with monosomy of chromosome 22 established from a human malignant meningioma that demonstrates *c-myc* amplification and can produce xenografts in nude mice.^116,364,365^ MN3 is another serially transplantable orthotopic cell model derived from a recurrent, malignant meningioma which has also been demonstrated to produce xenografts in nude mice while harboring several pathologic hallmarks of aggressive meningiomas including elevated Ki-67, vimentin expression, and *NF2* inactivation.^366^

One of the first benign meningioma cell lines, Ben-Men-1, was derived from a CNS WHO grade 1 meningioma by transducing tumor cells with the human *TERT* gene to overcome cellular senescence. However, while Ben-Men-1 proliferated rapidly in vitro, orthotopic xenografts using this cell line grew slowly, making it suboptimal for testing therapeutic agents.^367^ Furthermore, introducing alterations such as the human *TERT* gene or disruption of p53 and pRb pathways necessary to immortalize meningioma cell lines may also alter how closely these cell lines recapitulate their tumor of origin.^368^

To date, orthotopic xenograft models have been the gold standard for evaluating treatment efficacy in vivo, with tumor volumes readily evaluable by MRI or bioluminescence methods.^369,370^ However, these models are limited by the requirement for immunodeficient mice as hosts, which may be missing important immune cell populations for the study of tumor microenvironment interactions. Genetically engineered mouse models have attempted to overcome this shortcoming and different groups have leveraged conditional homozygous *NF2* knockout models, with and without *CDKN2A/B* loss or *SMO* activation, amongst other models. However, most of these models are hindered by similar limitations of prolonged tumor formation time, reduced animal survival due to secondary malignancies, low rates of induction, and complex time- and resource-intensive methodologies.^371,372^

Recently, 2 other novel ex vivo meningioma models have gained popularity: organotypic tumor slices and patient-derived spherical cell culture models (which include spheroids and organoids), both of which represent patient-derived 3D tumor models that closely recapitulate the genetic and epigenetic alterations of its parent tumor while also preserving cell type heterogeneity including immune and endothelial cells. While the former method enables meningioma tumor to remain in its original structure and within its native microenvironment, it requires larger amounts of intact tissue that cannot be damaged during sectioning and must account for the potential confounding effect of intratumoral heterogeneity within and between slices, making this technique challenging to standardize.^373,374^ Meningioma organoids, however, can be established from smaller amounts of tissue and can be readily multiplexed in order to perform rapid drug screening and other molecular assays such as DNA- or RNA-sequencing, flow cytometry, and immunohistochemistry.^375–378^ They can also be successfully established in 60–100% of cases from primary meningioma cells. Moreover, meningioma organoids and spheroids tend to express tumor markers such as progesterone receptor, more closely mimic the cell proliferation rate of human meningiomas compared to two-dimensional cultures, and may have increased transcriptomic markers for the epithelial-to-mesenchymal transition compared to traditional monolayer cultures.^379,380^ Variants identified in meningioma organoids such as *NF2* or *TRAF7* alterations are found at similar allele frequencies to their parental tumors, and the CNV profiles of the organoids also closely resemble those of the original tumor.^381^ The intratumoral heterogeneity and tumor microenvironment of the parental meningioma can also be recapitulated in organoid models, including retainment of immune cells (CD68 + macrophages, CD3 + T cells) and specific neoplastic cell subpopulations.^376^ Importantly, for organoids or spheroids, specific cell culture conditions need to be established in order to maintain the tumor microenvironment cells over time. While these innovations are promising, additional studies are needed to fully characterize both existing and novel preclinical meningioma models to assess their capability for replicating response to novel therapies.

## Future Clinical Trial Design and Other Future Directions

The design of meningioma clinical trials face several challenges largely related to tumor heterogeneity. One issue is that despite a growing body of evidence demonstrating the value of molecular classification systems, there has yet to be a unified molecular taxonomy that can be readily adopted by the WHO as standard of care classification. The ideal classification scheme should have a strong biological foundation and be readily implementable across most pathology laboratories. This standardization will ensure that patients are grouped based on clinically meaningful biological criteria rather than histopathology alone. Immunohistochemical correlates that can reliably identify molecular groups on a one-to-one basis would also be helpful, particularly in expanding trial access beyond tertiary referral centers in high-income countries that have access to sequencing technology. These measures may ultimately improve the uniformity of meningioma cases included in clinical trials treatment arms and decrease biologic heterogeneity that could confound treatment response, particularly amongst CNS WHO grade 2 cases that are a current cohort of interest in randomized trials.

In addition to classification, standardization of outcomes reporting is crucial.^151,382^ For instance, while PFS-6 has been considered the primary endpoint in most meningioma trials, this control benchmark is largely based on historical cases which may be graded differently today. To account for this, there is a need to retrospectively determine PFS-6 amongst molecularly defined meningioma cohorts, which would contribute to establishing a set of modern control cases for future prospective trial cases (which will likely also be molecularly driven), to benchmark against. Additionally, the relative improvement in PFS-6 that can be considered clinically meaningful should be standardized and these criteria will require subsequent validation. Lastly, as suggested by the RANO group, if PFS is to be used as the primary endpoint, neuroimaging over the last 6–12 months prior to trial enrollment is critical to establish a baseline rate of growth for comparison.^141^ Defining the appropriate interval and type of surveillance needed for each meningioma subgroup will also enable clinicians to detect tumor progression or recurrence at an early stage, leading to timely interventions and improved outcomes. Given the poor reliability of historical benchmarks such as PFS-6, ideally randomized trials should be conducted whenever possible. The implementation of adaptive clinical trial designs, which allow for real-time adjustments based on novel data, could also enhance the efficiency of clinical trials and accelerate the identification of novel treatments. Addressing these challenges through collaborative efforts among researchers, clinicians, and regulatory bodies will pave the way for more robust meningioma clinical trials, enhancing the precision and impact of future treatments.

## Summary

Meningiomas are the most common primary intracranial tumor type, and recent years have seen an increasing number of important discoveries that have shed light into their molecular underpinnings and heterogeneity. However, some unique groups of meningioma patients including those with *NF2*-associated schwannomatosis, RIMs, and pediatric patients have been largely excluded from most contemporary molecular studies. Furthermore, lack of access to sequencing resources and a relative dearth of high-fidelity preclinical models have also contributed to slower translation of encouraging benchtop findings to the bedside. While surgery and RT remain the only current standard of care options for patients, several promising systemic therapies have demonstrated evidence of efficacy in progressive or recurrent meningiomas with several important, ongoing clinical trials expected to report their results soon. Molecular profiling of meningiomas from these clinical trials including both responders and non-responders may uncover novel insights that can be leveraged for future study. Furthermore, in this current molecular era of meningioma research, there is a need to unify the many molecular classification schemes that have been discovered and utilize them to drive clinical trial design. Achieving this will require a data-driven approach and a consensus amongst experts in the field, including cooperation amongst those who originally developed these classification systems. This remains a high-priority goal of the cIMPACT-NOW working group and consortia such as ICOM and KAM. Implementation of a standardized molecular taxonomy for these tumors will ultimately serve as an important benchmark for future discovery and have a significant beneficial impact on the future care of patients with meningiomas.

## Supplementary material

Supplementary material is available online at *Neuro-Oncology* (https://academic.oup.com/neuro-oncology).

noae082_suppl_Supplementary_Table_S1
