# Supplementary material for: Meningioma: International Consortium on Meningiomas consensus review on scientific advances and treatment paradigms for clinicians, researchers, and patients
Source: Neuro Oncol. 2024 May 2;26(10):1742–80. doi: 10.1093/neuonc/noae082 (PMC11449035; doi:10.1093/neuonc/noae082)
Supplement: noae082_suppl_Supplementary_Table_S1 [file noae082_suppl_supplementary_table_s1.docx]

| PMCRT-MDA | MM-FAV | | | MM-UNFAV | | |  |  |  |  |  |  |  |  |  |  |  |  |
| --- | --- | --- | --- | --- | --- | --- | --- | --- | --- | --- | --- | --- | --- | --- | --- | --- | --- | --- |
|  | 1 | 2 | 3 | 1 | 2 | 3 |  |  |  |  |  |  |  |  |  |  |  |  |
|  | 79.5 | 20.5 | 0 | 57.0 | 35.5 | 7.5 |  |  |  |  |  |  |  |  |  |  |  |  |
| DKFZ | Ben-1 | | | Ben-2 | | | Ben-3 | | | Int-A | | | Int-B | | | Mal | | |
|  | 1 | 2 | 3 | 1 | 2 | 3 | 1 | 2 | 3 | 1 | 2 | 3 | 1 | 2 | 3 | 1 | 2 | 3 |
|  | 74.7 | 22.9 | 2.4 | 83.8 | 14.3 | 1.9 | 59.2 | 28.6 | 12.2 | 31.4 | 54.9 | 13.7 | 8.1 | 56.8 | 35.1 | 0 | 28.3 | 71.7 |
| Toronto | Immunogenic | | | *NF2*-Wildtype | | | Hypermetabolic | | | Proliferative | | |  |  |  |  |  |  |
|  | 1 | 2 | 3 | 1 | 2 | 3 | 1 | 2 | 3 | 1 | 2 | 3 |  |  |  |  |  |  |
|  | 90 | 10 | 0 | 65 | 30 | 5 | 50 | 40 | 10 | 10 | 50 | 40 |  |  |  |  |  |  |
| UCSF | Immune-Enriched | | | Merlin-Intact | | | Hypermitotic | | |  |  |  |  |  |  |  |  |  |
|  | 1 | 2 | 3 | 1 | 2 | 3 | 1 | 2 | 3 |  |  |  |  |  |  |  |  |  |
|  | 81.3 | 16.7 | 2.1 | 75.9 | 18.5 | 5.6 | 43.3 | 44.6 | 12.1 |  |  |  |  |  |  |  |  |  |
| Baylor | MenG B | | | MenG A | | | MenG C | | |  |  |  |  |  |  |  |  |  |
|  | 1 | 2 | 3 | 1 | 2 | 3 | 1 | 2 | 3 |  |  |  |  |  |  |  |  |  |
|  | 76.9 | 23.1 | 0 | 92.3 | 7.7 | 0 | 68.8 | 31.2 | 0 |  |  |  |  |  |  |  |  |  |

**Supplementary Table 1**. Relative proportion of meningiomas in each WHO grade belonging to each molecular/methylation class/group.
